# Supplementary material for: A genome-wide association study identifies genetic loci associated with specific lobar brain volumes
Source: Commun Biol. 2019 Aug 2;2:285. doi: 10.1038/s42003-019-0537-9 (PMC6677735; doi:10.1038/s42003-019-0537-9)
Supplement: Supplementary file 1 — Supplementary Information [file 42003_2019_537_MOESM1_ESM.pdf]

Supplementary methods, figures and acknowledgements

Supplementary Methods ... 2
Alzheimer's Disease Neuroimaging Initiative (ADNI): ... 2
Age, Gene/Environment Susceptibility-Reykjavik Study (AGES-Reykjavik) ... 4
Atherosclerosis Risk In Communities Study (ARIC) ... 6
Austrian Stroke Family Prevention Study (ASPS-Fam) ... 7
Cardiovascular Health Study (CHS) ... 8
Epidemiology of Dementia in Singapore (EDIS) ... 9
Erasmus Rucphen Family study (ERF) ... 10
Framingham Heart Study (FHS) ... 11
Genetic Study of Atherosclerosis Risk (GeneSTAR) ... 13
Leiden Longevity Study (LLS) ... 14
The Religious Orders Study and Memory and Aging Project (ROSMAP) ... 15
Rotterdam Study (RS-I, RS-II, RS-III) ... 16
The Saguenay Youth Study (SYS) ... 17
Three-City Dijon Study (3C-Dijon Study) ... 18
UK Biobank (UKBB) ... 20
Supplementary Figures ... 21
Acknowledgments ... 30
Study-specific acknowledgements ... 30
References ... 38

Supplementary Methods

Alzheimer's Disease Neuroimaging Initiative (ADNI):

Data used in the preparation of this article were obtained from the ADNI database (adni.loni.usc.edu). The ADNI was launched in 2003 by the National Institute on Aging (NIA), the National Institute of Biomedical Imaging and Bioengineering (NIBIB), the Food and Drug Administration (FDA), private pharmaceutical companies and non-profit organizations, as a $60 million, 5-year public-private partnership. The primary goal of ADNI has been to test whether serial magnetic resonance imaging (MRI), positron emission tomography (PET), other biological markers, and clinical and neuropsychological assessment can be combined to measure the progression of mild cognitive impairment (MCI) and early Alzheimer's disease (AD). Determination of sensitive and specific markers of very early AD progression is intended to aid researchers and clinicians to develop new treatments and monitor their effectiveness, as well as lessen the time and cost of clinical trials.

The Principal Investigator of this initiative is Michael W. Weiner, MD, VA Medical Center and University of California – San Francisco. ADNI is the result of efforts of many co-investigators from a broad range of academic institutions and private corporations, and subjects have been recruited from over 50 sites across the U.S. and Canada. The initial goal of ADNI was to recruit 800 subjects but ADNI has been followed by ADNI-GO and ADNI-2. To date these three protocols have recruited over 1500 adults, ages 55 to 90, to participate in the research, consisting of cognitively normal older individuals, people with early or late MCI, and people with early AD. The follow up duration of each group is specified in the protocols for ADNI-1, ADNI-2 and ADNI-GO. Subjects originally recruited for ADNI-1 and ADNI-GO had the option to be followed in ADNI-2. For up-to-date information, see www.adni-info.org. After excluding individuals with prevalent stroke and dementia at time of MRI-scanning, a total of 581 had phenotypic and genome-wide genotypic data in ADNI and 559 in ADNI-GO.

MRI protocol and phenotyping:

High-resolution structural brain MRI scans were acquired at 55 ADNI sites using 1.5T (ADNI1) or 3T MRI scanners (ADNI2GO) (GE Healthcare, Philips Medical Systems, or Siemens). GE scanners use inversion recovery-fast spoiled gradient recalled (IR-SPGR) sequences and Philips and Siemens use magnetization-prepared rapid gradient echo (MP-RAGE) sequences. Detailed MRI scanner protocols for T1-weighted sequences by vendor are available online (http://adni.loni.usc.edu/methods/documents/mri-protocols/). All T1-weighted images were segmented into supra-tentorial grey matter (GM), white matter (WM) and cerebrospinal fluid (CSF).

We used a previously described k-Nearest-Neighbor (kNN) algorithm, which was trained on six manually labeled atlases.[1] The volume GM and WM of both hemispheres was summed to calculate the volume of the four lobes. The volume GM and WM of both hemispheres was summed to calculate the volume of the four lobes.

Age, Gene/Environment Susceptibility-Reykjavik Study (AGES-Reykjavik)

The AGES cohort originally comprised a random sample of 30,795 men and women born in 1907–1935 and living in Reykjavik in 1967[2]. A total of 19381 attended, resulting in 71% recruitment rate. The study sample was divided into six groups by birth year and birth date within month. One group was designated for longitudinal follow-up and was examined in all stages. One group was designated a control group and was not included in examinations until 1991. Other groups were invited to participate in specific stages of the study. Between 2002 and 2006, the AGES-Reykjavik study re-examined 5764 survivors of the original cohort who had participated before in the Reykjavik Study. After excluding individuals with prevalent stroke and dementia at time of MRI-scanning, a total of 2478 had phenotypic and genome-wide genotypic data.

MRI protocol and phenotyping:

MR images were acquired on a single research-dedicated 1.5 T Signa Twinspeed EXCITE system (GE Medical Systems, Waukesha, WI) using a multi-channel phased array head cap coil. The structural image protocol included a T1-weighted three dimensional spoiled gradient echo (3D-SPGR) sequence (TE (time to echo), 8 ms; TR (time repetition), 21 ms; FA (flip angle), 30°; FOV (field of view), 240 mm; matrix, 256 × 256). Each volume consisted of 110 slices with 1.5 mm slice thickness, in-plane 0.94 x 0.94 mm. A proton density (PD)/T2 - weighted fast spin echo (FSE) sequence (TE1, 22 ms; TE2, 90 ms; TR, 3220 ms; echo train length, 8; FA, 90°; FOV, 220 mm; 256 × 256), and a fluid attenuated inversion recovery (FLAIR) sequence (TE, 100 ms; TR, 8000 ms, inversion time, 2000 ms, FA, 90°; FOV, 220 mm; matrix, 256 × 256). These latter two sequences were acquired with 3-mm thick slices and in-plane pixel size of 0.86 x 0.86 mm. All images were acquired to give full brain coverage and were localized at the AC/PC commissure line.

The lobar volumes were derived using a custom automatic image analysis pipeline, based on the Montreal Neurological Institute software. The pipeline was trained by an input of manually labelled images. It is based on the use of a probabilistic atlas consisting of a sample 314 individuals, an anatomical atlas consisting of an average of 4 individuals from the AGES-Reykjavik Study cohort and a multispectral tissue segmentation method. For the anatomical atlas the boundaries of the lobes were identified and drawn in axial, sagittal and coronal plane. Briefly, global brain tissue segmentation was first achieved: Stereotaxic registration was achieved following signal non-uniformity correction by an affine transformation of the T1-weighted images to the ICBM152 template. Tissue classification was achieved with an artificial neural network classifier. The absolute volumes of the four tissue types (GM, WM, WML and CSF) were subsequently calculated. The probabilistic atlas was non-linearly

warped to each subject, where total lobar volume (for each lobe) was obtained by counting the voxels belonging to each of the lobes from the atlas that overlap the subjects GM and WM masks[3, 4].

Atherosclerosis Risk In Communities Study (ARIC)

The ARIC study is a population-based cohort study of atherosclerosis and clinical atherosclerotic diseases[5]. At its inception (1987-1989), 15,792 men and women, including 11,478 white and 4,266 black participants were recruited from four U.S. communities: Suburban Minneapolis, Minnesota; Washington County, Maryland; Forsyth County, North Carolina; and Jackson, Mississippi. In the first 3 communities, the sample reflects the demographic composition of the community. In Jackson, only black residents were enrolled. Participants were between age 45 and 64 years at their baseline examination in 1987-1989 when blood was drawn for DNA extraction and participants consented to genetic testing. Vascular risk factors and outcomes, including transient ischemic attack, stroke and dementia, were determined in a standard fashion. During the first 2 years (1993-1994) of the third ARIC examination (V3), participants aged 55 and older from the Forsyth County and Jackson sites were invited to undergo cranial MRI. This subgroup of individuals with MRI scanning represents a random sample of the full cohort because examination dates were allocated at baseline through randomly selected induction cycles. After excluding individuals with prevalent stroke and dementia, a total of 413 white and 389 black participants had phenotypic and genome-wide genotypic data.

MRI protocol and phenotyping:

General Electric (General Electric Medical Systems) or Picker (Picker Medical Systems) 1.5-Tesla scanners were used for the MRI examination. [6] The scanning protocol included a series of sagittal T1-weighted scans and axial proton-density, T2-weighted and T1-weighted scans with 5 mm thickness and no interslice gaps. Images were interpreted directly from a PDS-4 digital workstation consisting of four 1024 X 1024-pixel monitors capable of displaying all 96 images simultaneously. Both ARIC and CHS used the same protocols for scanning and for interpretation.[7] All T1-weighted images were segmented into supra-tentorial grey matter (GM), white matter (WM) and cerebrospinal fluid (CSF). We used a previously described k-Nearest-Neighbor (kNN) algorithm, which was trained on six manually labeled atlases.[1] The volume GM and WM of both hemispheres was summed to calculate the volume of the four lobes.

Austrian Stroke Family Prevention Study (ASPS-Fam)

The ASPS-Fam is a prospective single-center community-based study on the cerebral effects of vascular risk factors in the normal aged population of the city of Graz, Austria.[8, 9] ASPS-Fam represents an extension of the ASPS, which was established in 1991.[10, 11] Between 2006 and 2013, study participants of the Austrian Stroke Prevention Study (ASPS) and their first-grade relatives were invited to enter ASPS-Fam. Inclusion criteria were no history of previous stroke or dementia and a normal neurologic examination. The study protocol was approved by the ethics committee of the Medical University of Graz, Austria, and written informed consent was obtained from all subjects. The entire cohort of 419 individuals underwent an extended diagnostic work- up including clinical history, blood tests, cognitive testing, and a thorough vascular risk factor assessment. Those 305 ASPS-Fam individuals who underwent MRI scanning and passed genotyping quality control were available for these analyses. They were all European Caucasians.

MRI protocol and phenotyping:

MRI scans for ASPS-Fam participants were obtained using a 3.0 T whole-body MR system (Tim Trio, Siemens, Erlangen) with a 12 channel head coil. The protocol included a high-resolution T1 weighted 3D sequence with magnetization prepared rapid gradient echo with whole brain coverage (repetition-time ¼ 1900ms,echo-time ¼ 2.19 ms, inversion-time ¼ 900 ms, flip angle ¼ 9, and isotropic resolution of 1mm) for assessing brain volume and for tissue segmentation. Volumetric segmentation was performed with the Freesurfer image analysis suite, version 5.2.0 (https://surfer.nmr.mgh.harvard.edu; Fischl, B., Salat, D.H., Busa, E., Albert, M., Dieterich, M., Haselgrove, C., van der Kouwe, A., Killiany, R., Kennedy, D., Klaveness, S., Montillo, A., Makris, N., Rosen, B., Dale, A.M., 2002. Whole brain segmentation: automated labeling of neuroanatomical structures in the human brain. Neuron 33, 341-355.) Total brain volume was calculated from the T1-weighted scans using the MRI post-processing software Freesurfer (https://surfer.nmr.mgh.harvard.edu). Volumes of the frontal, temporal, parietal and occipital lobes were defined as the sum of lobar cortical gray matter volumes (as defined in: https://surfer.nmr.mgh.harvard.edu/fswiki/CorticalParcellation) and the corresponding subcortical white matter volumes for each lobe.

Cardiovascular Health Study (CHS)

The CHS is a population-based cohort study of risk factors for coronary heart disease and stroke in adults ≥65 years conducted across four field centers.[12] The original predominantly European ancestry cohort of 5,201 persons was recruited in 1989-1990 from random samples of the Medicare eligibility lists; subsequently, an additional predominantly African-American cohort of 687 persons was enrolled for a total sample of 5,888. Blood samples were drawn from all participants at their baseline examination and DNA was subsequently extracted from available samples. Genotyping was performed at the General Clinical Research Center's Phenotyping/Genotyping Laboratory at Cedars-Sinai among CHS participants who consented to genetic testing and had DNA. European ancestry participants were excluded from the GWAS study sample due to the presence at study baseline of coronary heart disease, congestive heart failure, peripheral vascular disease, valvular heart disease, stroke or transient ischemic attack or lack of available DNA. Among those with successful GWAS, 2155 European ancestry and 507 African-American participants had available MRI scans for analysis. CHS was approved by institutional review committees at each field center and individuals in the present analysis had available DNA and gave informed consent including consent to use of genetic information for the study of cardiovascular disease.

MRI protocol and phenotyping:

Magnetic resonance imaging was performed on General Electric or Picker 1.5-Tesla scanners at 3 field centers and on a 0.35-Tesla Toshiba scanner at the fourth. The scanning protocol included a series of sagittal T1-weighted scans and axial proton-density, T2-weighted and T1-weighted scans with 5 mm thickness and no interslice gaps. Both ARIC and CHS used the same protocols for scanning and for interpretation [7] (see above for details). All T1-weighted images were segmented into supra-tentorial grey matter (GM), white matter (WM) and cerebrospinal fluid (CSF). We used a previously described k-Nearest-Neighbor (kNN) algorithm, which was trained on six manually labeled atlases.[1] The volume GM and WM of both hemispheres was summed to calculate the volume of the four lobes.

Epidemiology of Dementia in Singapore (EDIS)

The EDIS study draws subjects from the on-going population-based community-dwelling study of Chinese, Malays and Indians cohorts aged ≥40 years who participated in the Singapore Epidemiology of Eye Disease (SEED; n=10,033), which comprises the Singapore Chinese Eye Study (SCES; n=3,353), Singapore Malay Eye Study-2 (SiMES-2; n=3,280) and Singapore Indian Eye Study-2 (SINDI-2; n=3,400)[13]. As part of the baseline examinations in the SEED cohorts, genotyping was done in 2,587 SCES participants and 3,072 SiMES participants[14, 15]. In the present study we restricted analysis to the Chinese (EDIS-SCES) and Malay (EDIS-SiMES) component of EDIS, as the recruitment of the Indians has recently ended. Ethics approval for EDIS study was obtained from the Singapore Eye Research Institute (SERI) and National Healthcare Group Domain-Specific Review Board (DSRB). The study is being conducted in accordance with the Declaration of Helsinki. Written informed consent is obtained, in the preferred language of the participants, by bilingual study coordinators prior to their recruitment in the study. In the first phase of the EDIS Study, participants from SEED aged ≥ 60 years (n=1,538 Chinese and n=1,014 Malay) were screened using the 10-point Abbreviated Mental Test (AMT) and a self-report of progressive forgetfulness. Screen-positives were defined as AMT score ≤ 6, among those with ≤ 6 years of formal education, or ≤ 8 among those with > 6 years of formal education; or if the subject or caregiver reported progressive forgetfulness [yes/no]. A total of 300 Chinese and 308 Malay screen-positive subjects agreed to take part in the second phase of this study, which included an extensive neuropsychological test battery and brain MRI. Of these 131 Chinese and 211 Malay were included in the current analyses, who had genotyping and MRI data.

MRI protocol and phenotyping:

MRI was performed on a 3T Siemens Magnetom Trio Tim scanner, using a 32-channel head coil, at the Clinical Imaging Research Centre of the National University of Singapore. Subjects with claustrophobia, contraindications for MRI, or those who were unable to tolerate the procedure were excluded. All T1-weighted images were segmented into supra-tentorial grey matter (GM), white matter (WM) and cerebrospinal fluid (CSF). We used a previously described k-Nearest-Neighbor (kNN) algorithm, which was trained on six manually labeled atlases.[1] The volume GM and WM of both hemispheres was summed to calculate the volume of the four lobes.

Erasmus Rucphen Family study (ERF)

The Erasmus Rucphen Family (ERF) study is a family-based cohort study in a genetically isolated population from a community in the South-West of the Netherlands (Rucphen municipality) including 3000 participants. Participants are all descendants of a limited number of founders living in the 19th century, and all of Caucasian European descent. Extensive genealogical data is available for this population. The study population is described in detail elsewhere. As part of the protocol, genomic DNA was collected from all participants. Genotyping was done at the Human Genotyping Facility, Genetic Laboratory Department of Internal Medicine, Erasmus MC, Rotterdam, and at the Genotyping Center of Leiden University, The Netherlands. All participants gave informed consent and the study was approved by the medical ethics committee at Erasmus MC University Medical Center. In a follow-up analysis, 135 non-demented hypertensive (SBP ≥ 160, DBP ≥ 100 or use of antihypertensive medication) subjects aged 55-75 years were included for a new battery of tests including MRI scanning. Of these, 4 subjects were excluded because of physical constraints impeding the MRI scanning, and 2 subjects were excluded from analysis because large brain tumors were incidentally discovered.[16] Full genotype and phenotype data were available for 116 subjects after QC of the automated segmentations.

MRI protocol and phenotyping:

MRI scanning for the Erasmus Rucphen Family study was done on a 1.5 T MRI unit (GE Healthcare, Milwaukee, USA, Signa Excite software version 11×) fitted with a dedicated 8-channel head coil. The T1-weighted, proton density-weighted (PDw) and fluid-attenuated inversion recovery (FLAIR) sequences were used.[17] For the purpose of segmentation, the T1w scan is acquired in 3D at high in-plane resolution and with thin slices (voxel size < 1 mm3). [17] The scans were spatially registered using rigid registration. [17] Subsequently, the brain was extracted from the scan. Hereto a manually segmented brain mask, which excludes cerebellum, eyes and skull, was non-rigidly registered to the T1-weighted image using Elastix. [17] Finally, scans were corrected for intensity non-uniformity using the N3 method; non-uniformity correction was carried out within the brain mask. [17] All T1-weighted images were segmented into supra-tentorial grey matter (GM), white matter (WM) and cerebrospinal fluid (CSF). We used a previously described k-Nearest-Neighbor (kNN) algorithm, which was trained on six manually labeled atlases.[1] The volume GM and WM of both hemispheres was summed to calculate the volume of the four lobes.

Framingham Heart Study (FHS)

The FHS is a three-generation, single-site, community-based, ongoing cohort study initiated in 1948 to investigate the risk factors for cardiovascular disease. It now includes 3 generations of participants: the Original cohort followed since 1948;[18] their Offspring and spouses of the Offspring, followed since 1971;[19] and children from the largest Offspring families enrolled in 2000.[20] The Original cohort enrolled 5,209 men and women who comprised two-thirds of the adult population then residing in Framingham, MA. Survivors continue to receive biennial examinations. The Offspring cohort comprises 5,124 persons (including 3,514 biological offspring) and the Third-generation includes 4,095 participants with at least one parent in the Offspring Cohort; the Offspring and Third-generation cohorts are invited for follow-up examinations approximately once every 4 years.

The first two generations were invited to undergo an initial brain MRI in 1999-2005, and for the Third-generation, brain MRI began in 2009. The population of Framingham was virtually entirely white (Europeans of English, Scots, Irish and Italian descent) in 1948 when the Original cohort was recruited. Self-reports of ethnicity across all three generations were 99.7% whites, reflecting the ethnicity of the population of Framingham in 1948. All participants provided written informed consent at each examination. Study protocols and consent forms were approved by the Institutional Review Board of the Boston University Medical Center.

MRI protocol and phenotyping:

Removal of Non-brain tissues

The skull is removed using an atlas-based method[21] followed by human quality control to provide generally minor cleanup if needed. Structural MRI brain images are then nonlinearly registered performed by a cubic B-spline deformation [22] to a minimal deformation template (MDT) synthetic brain image[23] adapted for age range of 60 and above.

Image Intensity Inhomogeneity Correction

B1 field inhomogeneity is a common problem that limits the precision of image segmentation. We utilize a template-based iterative method for correcting field inhomogeneity bias.[24] At each algorithm iteration, the update of a B-spline deformation between an unbiased template image and the subject image is interleaved with estimation of a bias field based on the current template-to-image alignment. The bias field is modeled using a spatially smooth thin-plate spline interpolation based on ratios of local image patch intensity means between the deformed template and subject images. This is used to iteratively correct subject image intensities which are then used to improve the template-to-image deformation.

Gray, White and CSF Measurement

Our segmentation algorithm is based on an Expectation-Maximization (EM) algorithm that iteratively refines its segmentation estimates to produce outputs that are most consistent with the input intensities from the native-space T1 images along with a model of image smoothness.[25, 26] Like all EM algorithms, the system must be initialized with a reasonable estimate. We produce this initial estimate from the template-space warps of previously segmented images; because locations of WM/GM/CSF tissues are known in the template space, transforming these masks back to the each image's native space produces rough estimate 3-tissue segmentations. We then calculate the mean and standard deviation of the image intensities in locations labeled as each tissue type. These values then form the initial parameters for a Gaussian model of image intensity for each class. At each iteration, the algorithm uses a Gaussian model of T1-weighted image intensity for each tissue class, in order to produce a segmentation. In the first iteration, these models are estimated as described above. The segmentation yielded by these appearance models alone is then refined using a Markov Random Field (MRF) model, a computational statistical method that efficiently produces a label map consistent with both the input intensities and image smoothness statistics. Inference in the MRF is computed using an adaptive priors model.[26] This refined segmentation from the MRF is then used to compute new Gaussian intensity models for each tissue class, and the algorithm repeats, iteratively switching between calculating Gaussian appearance models and MRF-based segmentation, until convergence. The MRF-based segmentation at the final iteration is used as the final output segmentation. To calculate the volume of the four lobes the volume GM and WM of both hemispheres was summed

Genetic Study of Atherosclerosis Risk (GeneSTAR)

GeneSTAR (Genetic Study of Atherosclerosis Risk) is an ongoing prospective study designed to determine environmental, phenotypic, and genetic causes of premature cardiovascular disease. Participants (n=3533) were recruited from European- and African-American families (n=891) identified from 1983-2006 from probands with a premature coronary disease event prior to 60 years of age who were identified at the time of hospitalization in any of 10 Baltimore area hospitals. Apparently healthy siblings of the probands and offspring of the siblings and probands were screened for traditional coronary disease and stroke risk factors. A subset of this study population participated in an MRI study between 2009 and 2013. Siblings and offspring were excluded if they had a history of chronic corticosteroid use, life-threatening diseases, neurologic diseases that would preclude accurate MRI interpretation, and implanted metals that prohibited MRI scans. Participants with atrial fibrillation or symptomatic cardiovascular disease of any kind were excluded from the study.

MRI protocol and phenotyping:

MRI with a Philips 3T imaging unit was performed according to standardized protocols between 2009 and 2013. MPRAGE images were skull-stripped and co-registered to FLAIR images. Spatial normalization of the co-registered MPRAGE and FLAIR images into MNI space was performed via affine transformation. We segmented the brain in native MPRAGE space using an automated probabilistic methodology that employs a topology-preserving algorithm and mapped the resulting tissue mask to MNI space. We measured total brain, intracranial, cortical grey matter, and white matter volumes in native MPRAGE space. Intracranial volume was defined (in cubic millimeters) as the sum of all meningeal material, soft tissue, and sulcal and ventricular cerebrospinal volumes inferior to bone from the vertex to the foramen magnum. All T1-weighted images were segmented into supra-tentorial grey matter (GM), white matter (WM) and cerebrospinal fluid (CSF). We used a previously described k-Nearest-Neighbor (kNN) algorithm, which was trained on six manually labeled atlases.[1] The volume GM and WM of both hemispheres was summed to calculate the volume of the four lobes.

Leiden Longevity Study (LLS)

The Leiden Longevity Study (LLS) is a longitudinal cohort study consisting of 421 families of long-lived Caucasian siblings of Dutch descent together with their offspring and the partners thereof.[27] These partners were included as controls, since they have a comparable age and share the same socioeconomic and geographical background as the offspring. Families were recruited if at least two long-lived siblings were alive and aged at least 89 years for males and 91 years for females. These sex-specific age-criteria were used because of the higher life-expectancy for females compared to males.[27, 28] No selection criteria for health or demographic characteristics were applied. Recruitment took place between July 2002 and May 2006 and the families are followed up since that time.

MRI protocol and phenotyping:

All imaging was performed on a MRI system operating at a field strength of 3T (Philips Medical Systems, Best, the Netherlands). Three-dimensional T1-weighted images were acquired from all study participants with the following imaging parameters: TR = 9.7 ms, TE = 4.6 ms, FA = 8°, FOV = 224 × 177 × 168 mm, resulting in a nominal voxel size of 1.17 × 1.17 × 1.4 mm, covering the entire brain with no gap between slices, acquisition time was approximately five minutes. All T1-weighted images were segmented into supra-tentorial grey matter (GM), white matter (WM) and cerebrospinal fluid (CSF). We used a previously described k-Nearest-Neighbor (kNN) algorithm, which was trained on six manually labeled atlases.[1] The volume GM and WM of both hemispheres was summed to calculate the volume of the four lobes.

The Religious Orders Study and Memory and Aging Project (ROSMAP)

The Religious Orders Study (ROS), started in 1994, enrolled Catholic priests, nuns and brothers from about 40 groups in 12 states of the United States. The Rush Memory and Aging Project (MAP), started in 1997, enrolled older men and women from assisted living facilities in the Chicagoland area. Both are community based longitudinal clinical pathologic studies of aging and common chronic conditions of aging. Participants were free of known dementia at enrollment and agreed to annual clinical evaluations and organ donation after death. The studies were approved by the Institutional Review Board of Rush University Medical Center. Informed consent and a signed anatomical gift act were obtained from each participant.

MRI protocol and phenotyping:

Imaging substudies were initiated in 2009 in MAP and ROS. MRI scans were performed on a 1.5 Tesla scanner (General Electric, Waukesha, Wisconsin).[29] High-resolution T1-weighted anatomical data were obtained using 3D inversion-recovery fast spoiled gradient-echo (IR-FSPGR) sequence, with the following parameters: TE=2.8ms, TR=6.3ms, preparation time=1000ms, flip angle 8, field of view 24cm × 24cm, 160 sagittal slices, 1mm slice thickness, no gap, 224×192 image matrix reconstructed to 256×256. Two repetitions of the T1-weighted data acquired on each subject were co-registered and then averaged. Freesurfer (http://surfer.nmr.mgh.harvard.edu) was used for automatic segmentation. All Freesurfer results were reviewed and manually corrected when necessary.

Rotterdam Study (RS-I, RS-II, RS-III)

The Rotterdam Study is a population-based cohort study among inhabitants of a district of Rotterdam (Ommoord), The Netherlands, and aims to examine the determinants of disease and health in the elderly with a focus on neurogeriatric, cardiovascular, bone, and eye disease.36 In 1990-1993, 7,983 persons aged 55 years and older participated and were re-examined every 3 to 4 years (Rotterdam Study I). In 2000-2001 the cohort was expanded by 3,011 persons aged 55 and over who had not yet been part of the Rotterdam Study (Rotterdam Study II). In 2006-2008 a second expansion (Rotterdam Study III) of 3,932 persons aged 45 and over was realized. All participants had DNA extracted at their first visit. After excluding individuals with prevalent stroke and dementia at time of MRI-scanning, a total of 894 from RS-I, 1032 from RS-II and 2427 from RS-III had phenotypic and genome-wide genotypic data.

MRI protocol and phenotyping:

MRI scanning for the Rotterdam Study was done on a 1.5 T MRI unit (GE Healthcare, Milwaukee, USA, Signa Excite software version 11×) fitted with a dedicated 8-channel head coil. The T1-weighted, proton density-weighted (PDw) and fluid-attenuated inversion recovery (FLAIR) sequences were used.[17] For the purpose of segmentation, the T1w scan is acquired in 3D at high in-plane resolution and with thin slices (voxel size < 1 mm3). [17] The scans were spatially registered using rigid registration. [17] Subsequently, the brain was extracted from the scan. Hereto a manually segmented brain mask, which excludes cerebellum, eyes and skull, was non-rigidly registered to the T1-weighted image using Elastix. [17] Finally, scans were corrected for intensity non-uniformity using the N3 method; non-uniformity correction was carried out within the brain mask. [17] All T1-weighted images were segmented into supra-tentorial grey matter (GM), white matter (WM) and cerebrospinal fluid (CSF). We used a previously described k-Nearest-Neighbor (kNN) algorithm, which was trained on six manually labeled atlases.[1] The volume GM and WM of both hemispheres was summed to calculate the volume of the four lobes.

The Saguenay Youth Study (SYS)

The SYS is a multi-generational and multi-system cohort of a community-based sample of adolescents and their parents from the Saguenay Lac Saint Jean region (Quebec, Canada).[30] No individuals were excluded for prevalent stroke or prevalent dementia.

MRI protocol and phenotyping:

For adolescents, high-resolution anatomical T1-weighted (T1W) images were acquired in a Phillips 1.0-T superconducting magnet using the following parameters: 3D RF-spoiled gradient echo scan with 140-160 sagittal slices, 1-mm isotropic resolution, TR=25 ms, TE=5 ms, flip angle=30°.

For parents, T1W images were acquired in a Siemens 1.5T (Avanto) scanner using the 3D fast RF-spoiled gradient echo scan with 176 sagittal slices (1-mm isotropic resolution, TR = 2,400ms, TE = 2.65ms, TI = 1000ms, and flip angle = 8°).

For both adolescents and parents, the images were processed using an in-house image-processing pipeline employing the Minc Tool Kit (Montreal Neurological Institute:www.bic.mni.mcgill.ca/software/minc) and consisting of: (1) A correction for intensity inhomogeneities due to radio frequency field uniformity[31] and slice-wise intensity normalization using the median of slice-wise intensity ratios [32]; (2) Linear and nonlinear registration (using the ANTs algorithm[33]) to the ICBM152 template[34]; (3) Tissue classification using priors predefined on the ICBM152 template and projected back to the native space of each participant to enable a neural network-based classification of GM, WM and cerebrospinal fluid[35]; (4) Definition of all of the major lobes of the brain using a probabilistic atlas[36] warped (non-linearly) to each participant's native space; and (5) Estimates of the total brain volume and intra-cranial volume (ICV) using, respectively, brain and intracranial masks based on the ICBM152 template and warped (non-linearly) to each participant's native space. All steps of this processing pipeline were quality controlled (e.g., no movement artifacts, correct registration).

Total brain volume was obtained by assessing the volume within the brain mask. Total GM and WM were obtained by masking the tissue classification mask (step 3) with the brain mask (step 5). These values were then adjusted by ICV. Lobar volumes of GM and WM were obtained by masking tissue classification masks (step 3) with the respective lobar masks (step 4). To obtain relative lobar volumes, these values were adjusted by ICV. In total 1373 participants had both genotypes and brain lobar volume data available and were available for analysis.

Three-City Dijon Study (3C-Dijon Study)

The 3C study is a cohort study conducted in three French cities (Bordeaux, Dijon, and Montpellier), comprising 9,294 participants, designed to estimate the risk of dementia and cognitive impairment attributable to vascular factors[37]. Eligibility criteria included living in the city and being registered on the electoral rolls in 1999, 65 years or older, and not institutionalized. The study protocol was approved by the Ethical Committee of the University Hospital of Kremlin-Bicêtre and each participant signed an informed consent. Data reported in this article were obtained in Dijon (3C-Dijon study), where 4,931 individuals were recruited (1999–2001). The overall design of the 3C-Dijon study is detailed elsewhere.[37-39]

Participants aged less than 80 years and enrolled between June 1999 and September 2000 (n=2,763) were invited to undergo a brain MRI. Although 2,285 subjects agreed to participate (82.7%), because of financial limitations, 1,924 MRI scans were performed, of which 120 were not interpretable. DNA samples of 3C-Dijon participants were genotyped at the Centre National de Génotypage, Evry, France (www.cng.fr) with Illumina Human610-Quad® BeadChips.[40, 41] Lobar volume measures were available in 1,397 participants with genome-wide genotypes, after exclusion of participants with a diagnosis of dementia, stroke, or brain tumor at the time of MRI.

MRI image acquisition

MRI acquisition was performed on 1.5-Tesla Magnetom scanner (Siemens, Erlangen). Exclusion criteria for MRI were presence of an internal electrical or magnetic device; presence of metal fragments in the eyes, brain, or spinal cord; history of neurosurgery or aneurysm; and claustrophobia. A 3D high-resolution T1-weighted brain volume was acquired by using a 3D inversion recovery fast spoiled-gradient echo sequence (3D IR-SPGR; repetition time [TR] =97 msec; echo time [TE] = 4 msec; inversion time [TI] = 600 msec; coronal acquisition). The axially reoriented 3D volume matrix size was 256×192×256 with a 1.0× .98× .98 mm3 voxel size. T2- and proton density (PD)-weighted brain volumes were acquired by using a 2D dual spin-echo sequence with two echo times (TR=4400 msec; TE1=16 msec; TE2=98 msec). T2 and PD acquisitions consisted of 35 axial slices 3.5 mm thick (0.5 mm between slices spacing), with a 256×256 matrix size, and a 0.98×0.98 mm2 in-plane resolution.

Global brain and tissue volumetry

T1- and T2-weighted images from every participant were processed using a Voxel-Based Morphometry protocol based on SPM99[1] (http://www.fil.ion.ucl.ac.uk/spm/) that was modified to account for characteristics of aging brains. For each participant, gray matter (GM), white matter, and CSF volume were segmented and their respective volumes computed as the integral of voxel

intensities over the corresponding modulated tissue partition image. Total intracranial volume was computed as the sum of the three tissue volumes.

Lobar volumetry

As SPM does not provide lobar tissue volumes, ad'hoc procedures were implemented for estimating GM and WM lobar volumes for each individual.

First, we computed the GM volume of each ROI s of the AAL atlas[3] by integrating voxel intensities of the modulated grey matter density map within the ROI mask. Then each AAL ROI, except grey nuclei and cerebellar ROIs, was assigned to a specific lobe. GM lobar volume was then computed as the sum of the GM volumes of ROIs belonging to this lobe.

Since there is no anatomical limit of lobes within the white matter, we had to define a WM lobar parcellation scheme. This scheme was based on the GM lobar limits of the Talairach atlas. Specifically, we used oblique planes going through the Rolando sulcus, the Sylvian fissure, the parieto-occipital sulcus and the occipito-temporal sulcus for distinguishing between the occipital white matter of the different lobes. This scheme was applied to a white matter mask derived from a multi-modal segmentation (T1, T2 and PD)[4] and registered within the same standard space as the GM density map, for computing WM lobar volumes for each individual.

Eventually, frontal, temporal, parietal and occipital lobar volumes were computed as the sum of their GM and WM compartments.

UK Biobank (UKBB)

The UK Biobank (UKBB) is a large-scale epidemiological study of over 500,000 individuals aged 40-69 years from the United Kingdom (http://www.ukbiobank.ac.uk). The analyses presented here use data that were accessed via application 1155. Genetic data are available for the majority of these individuals[42] and as of 15 July 2017 13,269 of these participants had participated in a multimodal imaging sub-study [43, 44]. The genetic data used for these analyses uses only those variants imputed using the HRC reference panel. Imputation accuracy and allele frequency were recalculated in the subset of participants with imaging from the raw imputed data using HASE software, quality control filters used in the meta-analyses were applied to the UKBB data prior to analysis. To account for ethnicity, we included only subjects with white British ancestry (base on provided by UK Biobank information). To avoid correct cryptic relationship we excluded all subject with >=3rd degree of genetic relationship.

MRI protocol and phenotyping:

The analyses presented here use the lobar volume measurements of the 8,841 participants released by the UKBB. These volumes are derived from the T1 brain MRI, with extraction of these measures using FreeSurfer software version 6.0. No visual quality control of these data were performed (as the required files were not available for download). However, we removed outliers by setting data points more than 3 standard deviations from the mean to missing. For the calculation of the frontal lobe volume, we added up the grey matter volumes of the following regions: 'superiorfrontal', 'rostralmiddlefrontal', 'caudalmiddlefrontal', 'parsopercularis', 'parstriangularis', 'parsorbitalis', 'lateralorbitofrontal', 'medialorbitofrontal', 'precentral', 'paracentral' and 'frontalpole'. For temporal lobe volume, we summed the grey matter volumes of these regions: 'superiortemporal', 'middletemporal', 'inferiortemporal', 'bankssts', 'fusiform', 'transversetemporal', 'entorhinal', 'temporalpole' and 'parahippocampal'. For the parietal lobe, we used the grey matter volumes of 'superiorparietal', 'inferiorparietal', 'supramarginal', 'postcentral' and 'precuneus' regions. Finally, in order to calculate the occipital lobe volume we added up grey matter volumes of the following regions: 'lateraloccipital', 'lingual', 'cuneus' and 'pericalcarine'.

Supplementary Figures

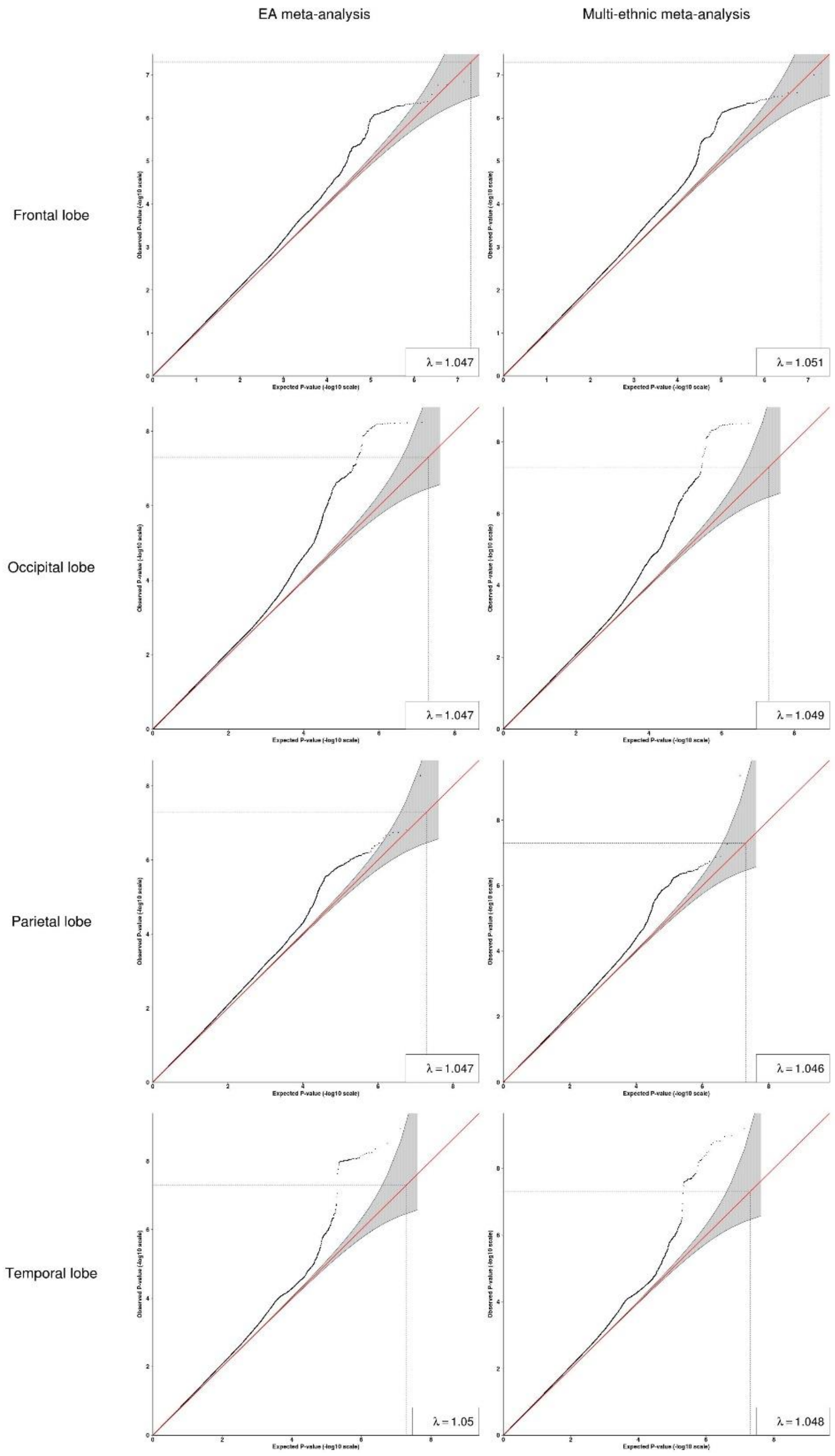

Supplementary Figure 1. Quantile-quantile (QQ) plot of four lobar volume genome-wide association study meta-analysis. The plots are shown for the European ancestry only and the multi-ethnic meta-analysis. All observed –log10 (p-values) are plotted against the expected –log10 (p-values) of a normal distribution. The grey lines depict the 95% confidence interval. The observed inflation was low ($\lambda < 1.05$) and was determined to be mainly a result of polygenicity using linkage disequilibrium score regression (intercept ≈ 1).[45]

1_155164480_OLV (A), I2 = 15.9(0.26)

study	N	MAF (A)	Effect	P-value
3C-Dijon	1397	0.482	0.06	0.825
ADNI	559	0.458	-0.59	0.187
ADNI2GO	581	0.458	-0.21	0.604
AGES	2478	0.429	-0.67	0.045
ARIC	416	0.457	-0.91	0.104
ARIC_blacks	405	0.321	0.51	0.351
ASPS	301	0.48	0.13	0.836
CHS	584	0.482	-1.28	0.005
EDIS_Chinese	131	0.241	-0.65	0.43
EDIS_Malays	211	0.37	0.12	0.897
ERF	116	0.531	-3.21	0.003
FHS	2079	0.48	-0.75	0.02
GENESTAR	441	0.474	-1	0.122
LLS	368	0.455	-0.6	0.253
RSI	894	0.479	-0.26	0.432
RSII	1032	0.469	-0.29	0.36
RSIII	2427	0.468	-0.64	0.003
RUSH1	137	0.42	-1.09	0.266
RUSH2	87	0.442	-1.34	0.26
SYS	1372	0.408	-0.51	0.105
**Meta-analysis: kNN**	**8165**	**0.456**	**-0.54**	**4.7e-06**
**Meta-analysis: others**	**7851**	**0.45**	**-0.42**	**4.6e-03**
**Meta-analysis**	**16016**	**0.455**	**-0.49**	**1.366e-07**

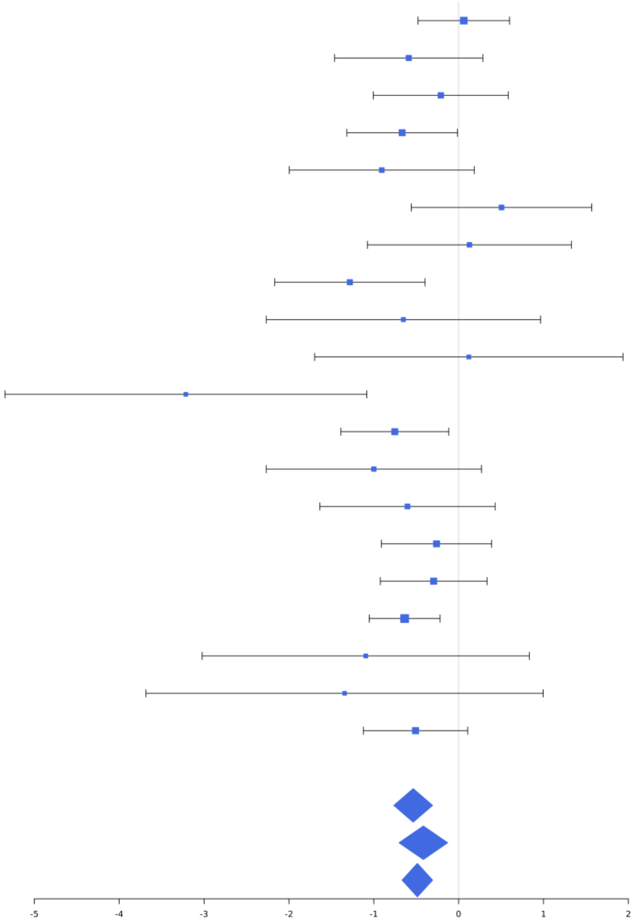

Supplementary Figure 2. Study-specific effects of rs12411216 or 1:155164480 on occipital lobar volume. Showing the effect for the allele A. Effects for the studies are shown as boxes (size according to sample size of the study) with 95% confidence intervals. As measure for heterogeneity the I^2 statistic (*p*-value) is shown. As a sensitivity analysis meta-analyses of only the studies using kNN-methods and those using other methods were performed.

3_147106319_PLV (T), I2 = 21.7(0.19)

study	N	MAF (T)	Effect	P-value
3C-Dijon	1397	0.183	0.81	0.204
ADNI	559	0.214	-0.34	0.682
ADNI2GO	581	0.214	-1.68	0.016
AGES	2478	0.226	-1.32	0.001
ARIC	416	0.213	-0.53	0.569
ARIC_blacks	405	0.252	-1.64	0.042
ASPS	301	0.244	-0.97	0.414
CHS	584	0.214	-0.66	0.4
EDIS_Chinese	131	0.336	-0.7	0.519
EDIS_Malays	211	0.352	-3.02	0.144
ERF	116	0.183	0.38	0.847
FHS	2081	0.212	-0.58	0.171
GENESTAR	441	0.202	-1.97	0.057
LLS	368	0.198	0.25	0.741
RSI	894	0.208	-1.02	0.089
RSII	1032	0.194	-1.44	0.011
RSIII	2427	0.201	-0.92	0.009
RUSH1	137	0.215	-4.58	0.024
RUSH2	87	0.19	-5.5	0.086
SYS	1369	0.166	-1.75	0.006
**Meta-analysis: kNN**	**8165**	**0.213**	**-0.99**	**4.8e-07**
**Meta-analysis: others**	**7850**	**0.204**	**-0.9**	**1.5e-04**
**Meta-analysis**	**16015**	**0.21**	**-0.95**	**4.362e-10**

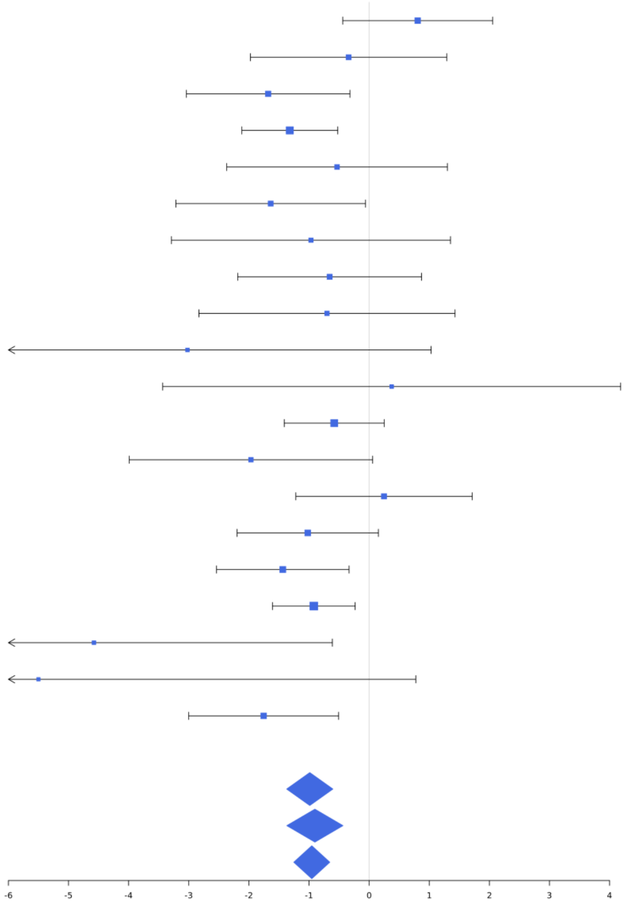

Supplementary Figure 3. Study-specific effects of rs2279829 or 3:147106319 on parietal lobar volume. Showing the effect for the allele T. Effects for the studies are shown as boxes (size according to sample size of the study) with 95% confidence intervals. As measure for heterogeneity the I^2 statistic (*p*-value) is shown. As a sensitivity analysis meta-analyses of only the studies using kNN-methods and those using other methods were performed.

4_1013382_OLV (A), I2 = 9.1(0.35)

study	N	MAF (A)	Effect	P-value
ADNI	559	0.185	-0.42	0.461
ADNI2GO	581	0.185	-1.45	0.011
AGES	2478	0.168	-0.54	0.275
ARIC	416	0.2	-0.84	0.301
ARIC_blacks	405	0.102	-1.34	0.232
ASPS	301	0.195	-0.1	0.909
CHS	584	0.212	-0.93	0.144
EDIS_Chinese	131	0.271	-0.8	0.391
EDIS_Malays	211	0.291	1.92	0.165
GENESTAR	441	0.189	-0.09	0.91
LLS	368	0.221	-0.15	0.833
RSI	894	0.205	-0.01	0.984
RSII	1032	0.208	-0.58	0.217
RSIII	2427	0.211	-1.36	0
RUSH1	137	0.162	-1.9	0.17
RUSH2	87	0.146	0.24	0.89
SYS	1372	0.215	-1.19	0.004
**Meta-analysis: kNN**	**8049**	**0.203**	**-0.81**	**3.8e-07**
**Meta-analysis: others**	**4375**	**0.184**	**-0.83**	**3.4e-03**
**Meta-analysis**	**12424**	**0.203**	**-0.82**	**6.169e-09**

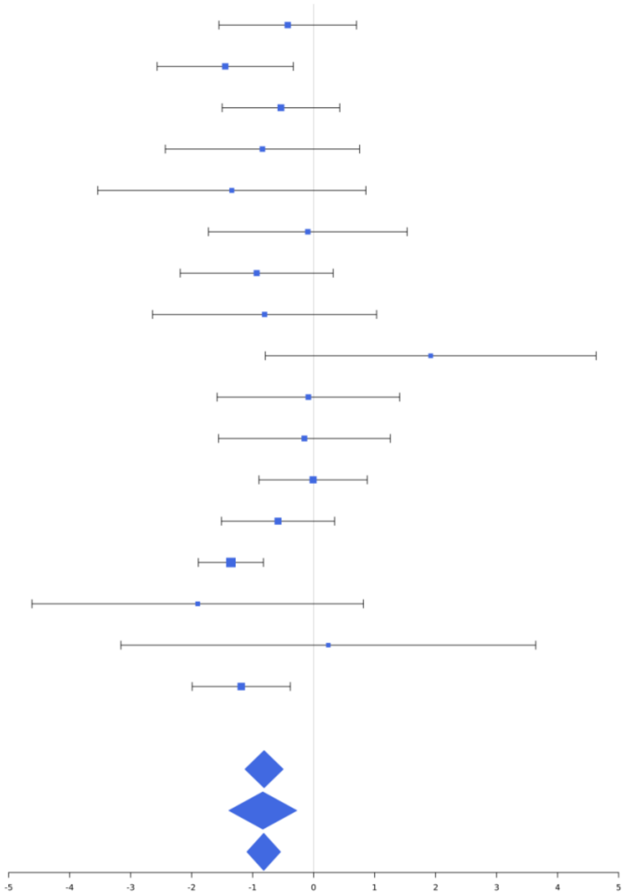

Supplementary Figure 4. Study-specific effects of rs74921869 or 4:1013382 on occipital lobar volume. Showing the effect for the allele A. Effects for the studies are shown as boxes (size according to sample size of the study) with 95% confidence intervals. As measure for heterogeneity the I^2 statistic (*p*-value) is shown. As a sensitivity analysis meta-analyses of only the studies using kNN-methods and those using other methods were performed.

6_126845380_OLV (A), I2 = 41.8(0.03)

study	N	MAF (A)	Effect	P-value
3C-Dijon	1397	0.237	-0.42	0.249
ADNI	559	0.235	-0.47	0.408
ADNI2GO	581	0.235	0.26	0.626
AGES	2478	0.199	-1.7	0
ARIC	416	0.234	-0.17	0.806
ARIC_blacks	405	0.208	-0.42	0.544
ASPS	301	0.229	-2.47	0.001
CHS	584	0.216	-0.93	0.119
EDIS_Chinese	131	0.476	-0.9	0.368
EDIS_Malays	211	0.414	-0.73	0.536
ERF	116	0.242	2.84	0.066
FHS	2079	0.241	-0.29	0.437
GENESTAR	441	0.252	-1.31	0.079
LLS	368	0.215	0.36	0.576
RSI	894	0.227	-0.75	0.064
RSII	1032	0.223	-0.07	0.865
RSIII	2427	0.224	-0.88	0.001
RUSH1	137	0.242	-2.92	0.016
RUSH2	87	0.224	0.88	0.506
SYS	1372	0.219	-0.86	0.034
**Meta-analysis: kNN**	**8165**	**0.235**	**-0.5**	**7.1e-04**
**Meta-analysis: others**	**7851**	**0.223**	**-0.87**	**2.3e-06**
**Meta-analysis**	**16016**	**0.232**	**-0.64**	**3.976e-08**

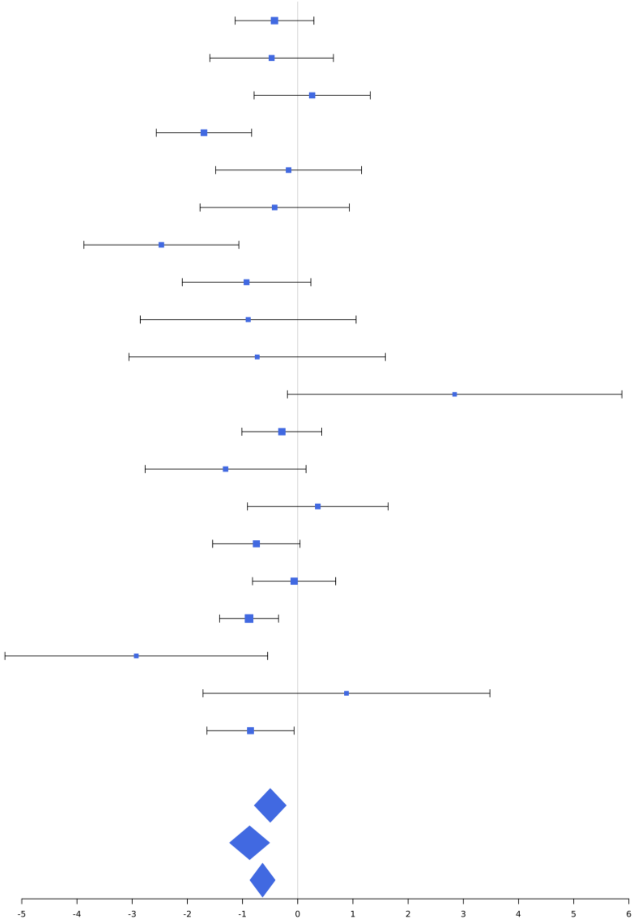

Supplementary Figure 5. Study-specific effects of rs1337736 or 6:126845380 on occipital lobar volume. Showing the effect for the allele A. Effects for the studies are shown as boxes (size according to sample size of the study) with 95% confidence intervals. As measure for heterogeneity the I^2 statistic (*p*-value) is shown. As a sensitivity analysis meta-analyses of only the studies using kNN-methods and those using other methods were performed.

12_65793942_TLV (A), I2 = 38.2(0.05)

study	N	MAF (A)	Effect	P-value
3C-Dijon	1397	0.395	0.34	0.377
ADNI	559	0.398	1.63	0.012
ADNI2GO	581	0.398	0.4	0.495
AGES	2478	0.37	1.51	0
ARIC	416	0.37	0.88	0.273
ARIC_blacks	405	0.292	1.02	0.174
ASPS	301	0.384	-0.82	0.315
CHS	584	0.344	1.77	0
EDIS_Chinese	131	0.557	0.72	0.549
EDIS_Malays	211	0.646	-0.68	0.594
ERF	116	0.424	-0.08	0.964
FHS	2077	0.389	1.05	0.001
GENESTAR	441	0.37	0.73	0.238
LLS	368	0.349	0.37	0.478
RSI	894	0.353	0.1	0.815
RSII	1032	0.362	0.73	0.057
RSIII	2427	0.35	0.16	0.509
SYS	1371	0.357	0.7	0.156
**Meta-analysis: kNN**	**8165**	**0.369**	**0.54**	**1.2e-04**
**Meta-analysis: others**	**7624**	**0.378**	**0.91**	**2.2e-07**
**Meta-analysis**	**15789**	**0.37**	**0.68**	**6.427e-10**

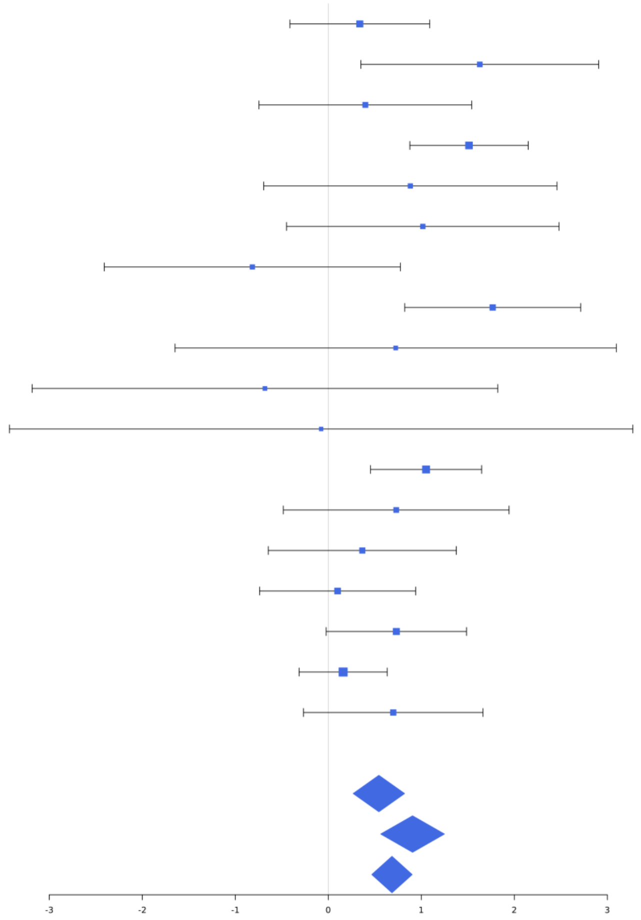

Supplementary Figure 6. Study-specific effects of rs147148763 or 12: 65793942 on temporal lobar volume. Showing the effect for the allele A. Effects for the studies are shown as boxes (size according to sample size of the study) with 95% confidence intervals. As measure for heterogeneity the I^2 statistic (*p*-value) is shown. As a sensitivity analysis meta-analyses of only the studies using kNN-methods and those using other methods were performed.

14_59631075_OLV (G), I2 = 34.5(0.09)

study	N	MAF (G)	Effect	P-value
3C-Dijon	1397	0.131	0.27	0.528
ADNI	559	0.121	-0.6	0.39
ADNI2GO	581	0.121	-1.46	0.025
AGES	2478	0.111	-0.73	0.167
ARIC	416	0.129	-0.74	0.372
ARIC_blacks	405	0.02	-3.45	0.057
ASPS	301	0.124	-2.28	0.012
CHS	584	0.117	-0.85	0.245
EDIS_Malays	211	0.033	-0.65	0.81
ERF	116	0.117	-0.53	0.772
FHS	2079	0.137	-0.95	0.014
LLS	368	0.14	1.47	0.06
RSI	894	0.133	-1.2	0.015
RSII	1032	0.126	-1.18	0.015
RSIII	2427	0.131	-1.12	0
SYS	1372	0.131	-1.03	0.027
**Meta-analysis: kNN**	**7593**	**0.119**	**-0.96**	**2.7e-07**
**Meta-analysis: others**	**7627**	**0.126**	**-0.7**	**1.2e-03**
**Meta-analysis**	**15220**	**0.128**	**-0.85**	**2.89e-09**

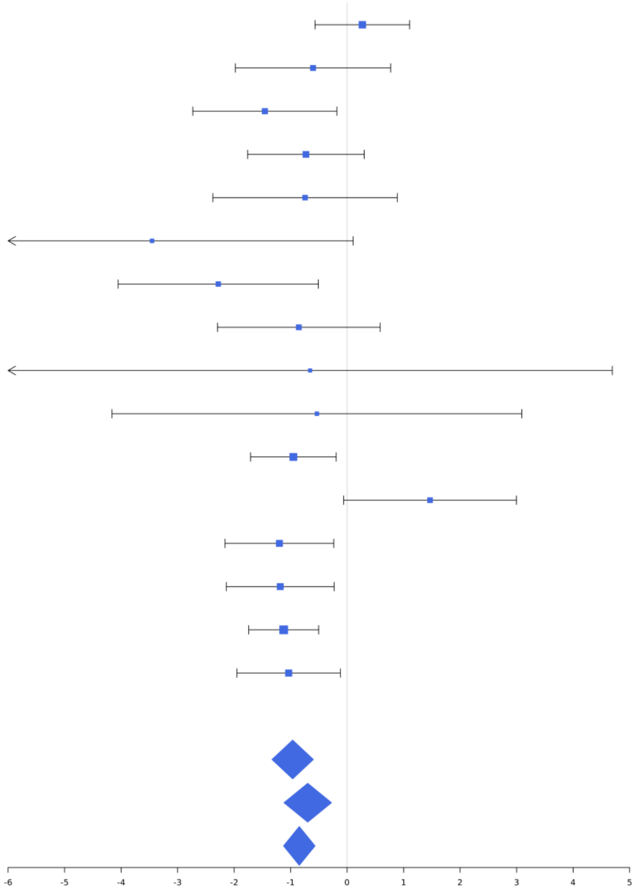

Supplementary Figure 7. Study-specific effects of rs1337736 or 14:59631075 on occipital lobar volume. Showing the effect for the allele G. Effects for the studies are shown as boxes (size according to sample size of the study) with 95% confidence intervals. As measure for heterogeneity the I^2 statistic (*p*-value) is shown. As a sensitivity analysis meta-analyses of only the studies using kNN-methods and those using other methods were performed.

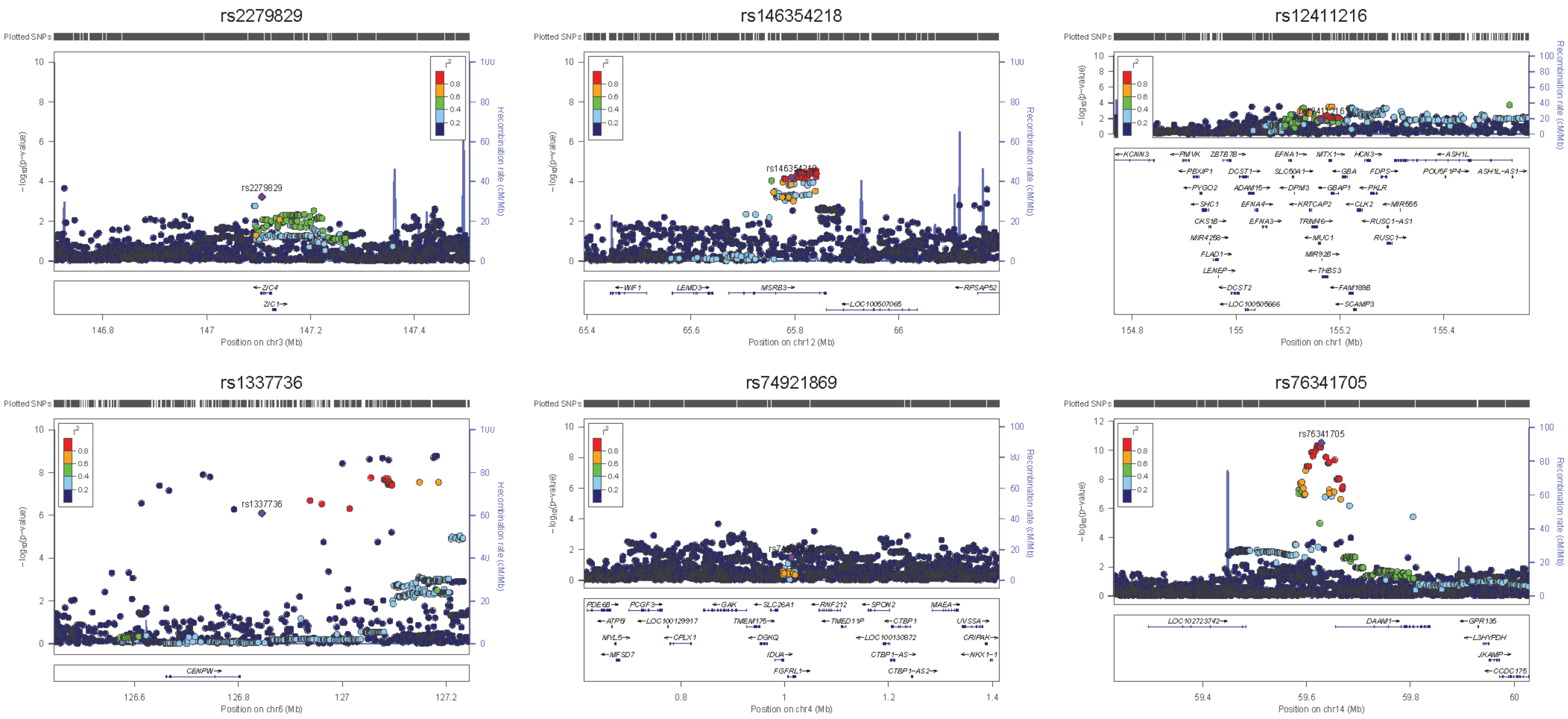

Supplementary Figure 8. Regional view of the genome-wide significant loci in the replication sample. For each panel, zoomed in Manhattan plots (± 400 kb from top SNP) are shown with gene models below (refFlat UCSC build 19). Each plot was made using the LocusZoom software (http://locuszoom.org/). For the locus containing genetic variant rs147148763, we used another genome-wide significant variant in the same locus instead (rs76341705).

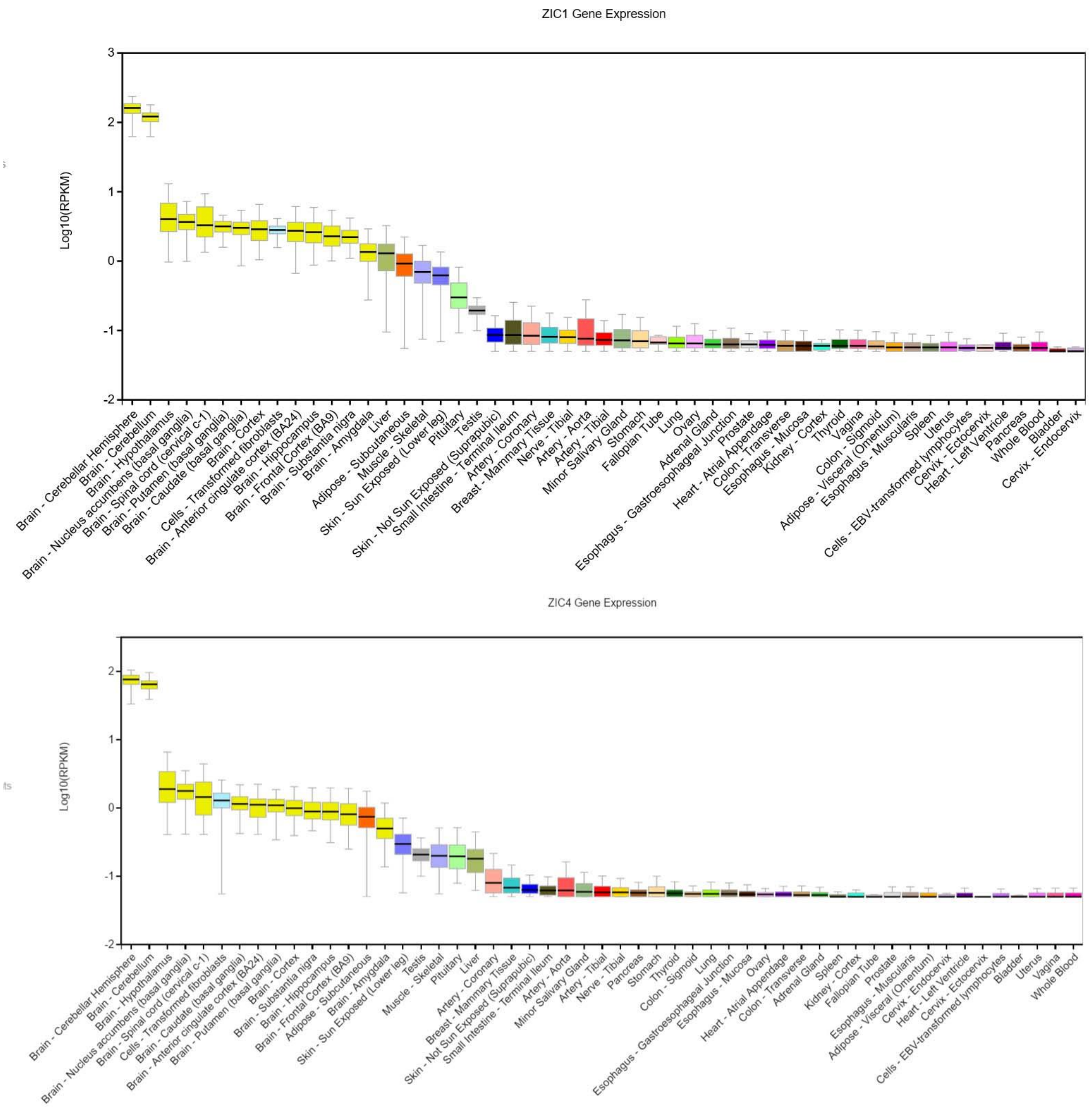

Supplementary Figure 9. GTEx expression of *ZIC1* and *ZIC4*.

Acknowledgments

CHARGE: Infrastructure for the CHARGE Consortium is supported in part by the National Heart, Lung, and Blood Institute grant HL105756 and for the NeuroCHARGE phenotype working group through the National Institute on Aging grant AG033193.

Study-specific acknowledgements

Alzheimer's Disease Neuroimaging Initiative (ADNI): Data collection and sharing for this project was funded by the ADNI (National Institutes of Health Grant U01 AG024904) and DODADNI (Department of Defense award number W81XWH-12-2-0012). ADNI is funded by the National Institute on Aging, the National Institute of Biomedical Imaging and Bioengineering, and through generous contributions from the following: Alzheimer’s Association; Alzheimer’s Drug Discovery Foundation; BioClinica, Inc.; Biogen Idec Inc.; Bristol-Myers Squibb Company; Eisai Inc.; Elan Pharmaceuticals, Inc.; Eli Lilly and Company; F. Hoffmann-La Roche Ltd and its affiliated company Genentech, Inc.; GE Healthcare; Innogenetics, N.V.; IXICO Ltd.; Janssen Alzheimer Immunotherapy Research & Development, LLC.; Johnson & Johnson Pharmaceutical Research & Development LLC.; Medpace, Inc.; Merck & Co., Inc.; Meso Scale Diagnostics, LLC.; NeuroRx Research; Novartis Pharmaceuticals Corporation; Pfizer Inc.; Piramal Imaging; Servier; Synarc Inc.; and Takeda Pharmaceutical Company. The Canadian Institutes of Health Research is providing funds to support ADNI clinical sites in Canada. Private sector contributions are facilitated by the Foundation for the National Institutes of Health (www.fnih.org). The grantee organization is the Northern California Institute for Research and Education, and the study is coordinated by the Alzheimer's Disease Cooperative Study at the University of California, San Diego. ADNI data are disseminated by the Laboratory of Neuro Imaging at the University of Southern California.

Data used in preparing this article were obtained from the Alzheimer’s Disease Neuroimaging Initiative (ADNI) database (adni.loni.usc.edu). As such, many investigators within the ADNI contributed to the design and implementation of ADNI and/or provided data but did not participate in analysis or writing of this report. A complete listing of ADNI investigators can be found at: http://adni.loni.usc.edu/wp-content/uploads/how_to_apply/ADNI_Acknowledgement_List.pdf: Michael Weiner (UC San Francisco), Paul Aisen (UC San Diego), Ronald Petersen (Mayo Clinic, Rochester), Clifford R. Jack, Jr. (Mayo Clinic, Rochester), William Jagust (UC Berkeley), John Q. Trojanowki (U Pennsylvania), Arthur W. Toga (USC), Laurel Beckett (UC Davis), Robert C. Green

(Brigham and Women's Hospital / Harvard Medical School), Andrew J. Saykin (Indiana University), John Morris (Washington University St. Louis), Leslie M. Shaw (University of Pennsylvania); ADNI External Advisory Board (ESAB): Zaven Khachaturian (Prevent Alzheimer's Disease 2020), Greg Sorensen (Siemens), Maria Carrillo (Alzheimer's Association), Lew Kuller (University of Pittsburgh), Marc Raichle (Washington University St. Louis), Steven Paul (Cornell University), Peter Davies (Albert Einstein College of Medicine of Yeshiva University), Howard Fillit (AD Drug Discovery Foundation), Franz Hefti (Acumen Pharmaceuticals), Davie Holtzman (Washington University St. Louis), M. Marcel Mesulman (Northwestern University), William Potter (National Institute of Mental Health), Peter Snyder (Brown University); **ADNI 2 Private Partner Scientific Board (PPSB) Chair:** Adam Schwartz (Eli Lilly); **Data and Publication Committee (DPC)**: Robert C. Green (Brigham and Women's Hospital/Harvard Medical School (Chair)); **Resource Allocation Review Committee**: Tom Montine (University of Washington (Chair)); **Clinical Core Leaders:** Ronald Petersen (Mayo Clinic, Rochester), Paul Aisen (UC San Diego); **Clinical Informatics and Operations**: Ronald G. Thomas (UC San Diego), Michael Donohue (UC San Diego), Sarah Walter (UC San Diego), Devon Gessert (UC San Diego), Tamie Sather (UC San Diego), Gus Jiminez (UC San Diego); **Biostatistics Core Leaders and Key Personnel:** Laurel Beckett (UC Davis), Danielle Harvey (UC Davis), Michael Donohue (UC San Diego); **MRI Core Leaders and Key Personnel:** Clifford R. Jack, Jr. (Mayo Clinic, Rochester), Matthew Bernstein (Mayo Clinic, Rochester), Nick Fox (University of London), Paul Thompson (Keck School of Medicine of USC), Norbert Schuff (UCSF), Charles DeCarli (UC Davis), Bret Borowski (Mayo Clinic), Jeff Gunter (Mayo Clinic), Matt Senjem (Mayo Clinic), Prashanthi Vemuri (Mayo Clinic), David Jones (Mayo Clinic), Kejal Kantarci (Mayo Clinic), Chad Ward (Mayo Clinic); **PET Core Leaders and Key Personnel:** William Jagust (UC Berkeley), Robert A. Koeppe (University of Michigan), Norm Foster (University of Utah), Eric M. Reiman (Banner Alzheimer's Institute), Kewei Chen (Banner Alzheimer's Institute), Chet Mathis (University of Pittsburgh), Susan Landau (UC Berkeley); **Neuropathology Core Leaders:** John Morris (Washington University St. Louis), Nigel J. Cairns (Washington University St. Louis), Erin Householder (Washington University St. Louis), Lisa Taylor-Reinwald (Washington University St. Louis); **Biomarkers Core Leaders and Key Personnel:** J.Q. Trojanowki (UPenn School of Medicine), Les Shaw (UPenn School of Medicine), Virginia M.Y. Lee (UPenn School of Medicine), Magdalena Korecka (UPenn School of Medicine), Michal Figurski (UPenn School of Medicine); **Informatics Core Leaders and Key Personnel:** Arthur W. Toga (USC), Karen Crawford (USC), Scott Neu (USC); **Genetics Core Leaders and Key Personnel:** Andrew J. Saykin (Indiana University), Tatiana M. Foroud (Indiana University), Steven Potkin (UC Irvine), Li Shen (Indiana University), Kelley Faber (Indiana University), Sungeun Kim (Indiana University), Kwangsik Nho (Indiana University); **Initial Concept Planning & Development:** Michael W. Weiner (UC San Francisco), Leon Thal (UC San Diego), Zaven Khachaturian (Prevent Alzheimer's Disease 2020); **Early Project Development:** Zaven Khachaturian (Prevent

Alzheimer's Disease 2020), Richard Frank (General Electric), Peter J. Snyder (University of Connecticut), Michael W. Weiner (UC San Francisco), Leon Thal (UC San Diego), Neil Buckholtz (NIA), William Potter (NIMH), Steven Paul (Cornell University), Marilyn Albert (The Johns Hopkins University); **NIA:** John Hsiao (National Institute on Aging/National Institutes of Health).
The full ADNI investigator lists includes: **Oregon Health and Science University:** Jeffrey Kaye, Joseph Quinn, Betty Lind, Raina Carter, Sara Dolen – Past Investigator; **University of Southern California:** Boris A. Gutman, Lon S. Schneider, Sonia Pawluczyk, Mauricio Beccera, Liberty Teodoro, Bryan M. Spann, DO – Past Investigator; **University of California-San Diego:** James Brewer, Helen Vanderswag, Adam Fleisher – Past Investigator; **University of Michigan:** Judith L. Heidebrink, Joanne L. Lord; **Mayo Clinic, Rochester:** Ronald Petersen, Sara S. Mason, Colleen S. Albers, David Knopman, Kris Johnson – Past Investigator; **Baylor College of Medicine:** Rachelle S. Doody, Javier Villanueva-Meyer, Munir Chowdhury, Susan Rountree, Mimi Dang; **Columbia University Medical Center:** Yaakov Stern, Lawrence S. Honig, Karen L. Bell; **Washington University, St. Louis:** Beau Ances, John C. Morris, Maria Carroll, Sue Leon, Erin Householder, Mark A. Mintun – Past Investigator, Stacy Schneider – Past Investigator, Angela Oliver – Past Investigator; **University of Alabama - Birmingham:** Daniel Marson, Randall Griffith, David Clark, David Geldmacher, John Brockington, Erik Roberson; **Mount Sinai School of Medicine:** Hillel Grossman, Effie Mitsis; **Rush University Medical Center:** Leyla deToledo-Morrell, Raj C. Shah; **Wien Center:** Ranjan Duara, Daniel Varon, Maria T. Greig, Peggy Roberts– Past Investigator; **Johns Hopkins University:** Marilyn Albert, Chiadi Onyike, Daniel D'Agostino II, Stephanie Kielb – Past Investigator; **New York University:** James E. Galvin, Dana M. Pogorelec, Brittany Cerbone, Christina A. Michel, Henry Rusinek – Past Investigator, Mony J de Leon – Past Investigator, Lidia Glodzik – Past Investigator, Susan De Santi – Past Investigator; **Duke University Medical Center:** P. Murali Doraiswamy, Jeffrey R. Petrella, Terence Z. Wong; **University of Pennsylvania:** Steven E. Arnold, Jason H. Karlawish, David Wolk; **University of Kentucky:** Charles D. Smith, Greg Jicha, Peter Hardy, Partha Sinha, Elizabeth Oates, Gary Conrad; **University of Pittsburgh:** Oscar L. Lopez, MaryAnn Oakley, Donna M. Simpson; **University of Rochester Medical Center:** Anton P. Porsteinsson, Bonnie S. Goldstein, Kim Martin, Kelly M. Makino – Past Investigator, M. Saleem Ismail – Past Investigator, Connie Brand – Past Investigator; **University of California, Irvine:** Ruth A. Mulnard, Gaby Thai, Catherine Mc-Adams-Ortiz; **University of Texas Southwestern Medical School:** Kyle Womack, Dana Mathews, Mary Quiceno, Ramon Diaz-Arrastia – Past Investigator, Richard King – Past Investigator, Myron Weiner – Past Investigator, Kristen Martin-Cook – Past Investigator, Michael DeVous – Past Investigator; **Emory University:** Allan I. Levey, James J. Lah, Janet S. Cellar; **University of Kansas, Medical Center:** Jeffrey M. Burns, Heather S. Anderson, Russell H. Swerdlow; **University of California, Los Angeles:** Liana Apostolova, Kathleen Tingus, Ellen Woo, Daniel H.S. Silverman, Po H. Lu – Past Investigator, George Bartzokis – Past Investigator; **Mayo Clinic, Jacksonville:** Neill R Graff-Radford,

Francine Parfitt, Tracy Kendall, Heather Johnson – Past Investigator; **Indiana University:** Martin R. Farlow, Ann Marie Hake, Brandy R. Matthews, Scott Herring, Cynthia Hunt; **Yale University School of Medicine:** Christopher H. van Dyck, Richard E. Carson, Martha G. MacAvoy; **McGill Univ., Montreal-Jewish General Hospital:** Howard Chertkow, Howard Bergman, Chris Hosein; **Sunnybrook Health Sciences, Ontario:** Sandra Black, Dr Bojana Stefanovic, Curtis Caldwell; **U.B.C. Clinic for AD & Related Disorders:** Ging-Yuek Robin Hsiung, Howard Feldman, Benita Mudge, Michele Assaly, – Past Investigator; **Cognitive Neurology - St. Joseph's, Ontario:** Andrew Kertesz, John Rogers, Dick Trost; **Cleveland Clinic Lou Ruvo Center for Brain Health:** Charles Bernick, Donna Munic; **Northwestern University:** Diana Kerwin, Marek-Marsel Mesulam, Kristine Lipowski, Chuang-Kuo Wu – Past Investigator, Nancy Johnson – Past Investigator; **Premiere Research Inst (Palm Beach Neurology):** Carl Sadowsky, Walter Martinez, Teresa Villena; **Georgetown University Medical Center:** Raymond Scott Turner, Kathleen Johnson, Brigid Reynolds; **Brigham and Women's Hospital:** Reisa A. Sperling, Keith A. Johnson, Gad Marshall, Meghan Frey – Past Investigator; **Stanford University:** Jerome Yesavage, Joy L. Taylor, Barton Lane, Allyson Rosen – Past Investigator, Jared Tinklenberg – Past Investigator; **Banner Sun Health Research Institute:** Marwan N. Sabbagh, Christine M. BeldenSandra A. Jacobson, Sherye A. Sirrel; **Boston University:** Neil Kowall, Ronald Killiany, Andrew E. Budson, Alexander Norbash – Past Investigator, Patricia Lynn Johnson – Past Investigator; **Howard University:** Thomas O. Obisesan, Saba Wolday, Joanne Allard; **Case Western Reserve University:** Alan Lerner, Paula Ogrocki, Leon Hudson – Past Investigator; **University of California, Davis – Sacramento:** Evan Fletcher, Owen Carmichael, John Olichney, Charles DeCarli – Past Investigator; **Neurological Care of CNY:** Smita Kittur; **Parkwood Hospital:** Michael Borrie, T-Y Lee, Dr Rob Bartha; **University of Wisconsin:** Sterling Johnson, Sanjay Asthana, Cynthia M. Carlsson; **University of California, Irvine - BIC:** Steven G. Potkin, Adrian Preda, Dana Nguyen; **Banner Alzheimer's Institute:** Pierre Tariot, Adam Fleisher, Stephanie Reeder; **Dent Neurologic Institute:** Vernice Bates, Horacio Capote, Michelle Rainka; **Ohio State University:** Douglas W. Scharre, Maria Kataki, Anahita Adeli; **Albany Medical College:** Earl A. Zimmerman, Dzintra Celmins, Alice D. Brown; **Hartford Hospital, Olin Neuropsychiatry Research Center:** Godfrey D. Pearlson, Karen Blank, Karen Anderson; **Dartmouth-Hitchcock Medical Center:** Robert B. Santulli, Tamar J. Kitzmiller, Eben S. Schwartz – Past Investigator; **Wake Forest University Health Sciences:** Kaycee M. Sink, Jeff D. Williamson, Pradeep Garg, Franklin Watkins – Past Investigator; **Rhode Island Hospital:** Brian R. Ott, Henry Querfurth, Geoffrey Tremont; **Butler Hospital:** Stephen Salloway, Paul Malloy, Stephen Correia; **UC San Francisco:** Howard J. Rosen, Bruce L. Miller; **Medical University South Carolina:** Jacobo Mintzer, Kenneth Spicer, David Bachman; **St. Joseph’s Health Care:** Elizabether Finger, Stephen Pasternak, Irina Rachinsky, John Rogers, Andrew Kertesz – Past Investigator, Dick Drost – Past Investigator; **Nathan Kline Institute:** Nunzio Pomara, Raymundo Hernando, Antero Sarrael; **University of Iowa**

College of Medicine: Susan K. Schultz, Laura L. Boles Ponto, Hyungsub Shim, Karen Elizabeth Smith; **Cornell University:** Norman Relkin, Gloria Chaing, Lisa Raudin; **University of South Florida: USF Health Byrd Alzheimer's Institute:** Amanda Smith, Kristin Fargher, Balebail Ashok Raj.

Age, Gene/Environment Susceptibility-Reykjavik Study (AGES): This study has been funded by NIH contract N01-AG-1-2100, the NIA Intramural Research Program, Hjartavernd (the Icelandic Heart Association), and the Althingi (the Icelandic Parliament). The study is approved by the Icelandic National Bioethics Committee, VSN: 00–063. The researchers are indebted to the participants for their willingness to participate in the study.

Atherosclerosis Risk in Communities (ARIC) Study: The Atherosclerosis Risk in Communities study was performed as a collaborative study supported by National Heart, Lung, and Blood Institute (NHLBI) contracts (HHSN268201100005C, HSN268201100006C, HSN268201100007C, HHSN268201100008C, HHSN268201100009C, HHSN268201100010C, HHSN268201100011C, and HHSN268201100012C), R01HL70825, R01HL087641, R01HL59367, and R01HL086694; National Human Genome Research Institute contract U01HG004402; and National Institutes of Health (NIH) contract HHSN268200625226C. Infrastructure was partly supported by grant No. UL1RR025005, a component of the NIH and NIH Roadmap for Medical Research. This project was partially supported by National Institutes of Health R01 grants HL084099 and NS087541 to MF.

Austrian Stroke Prevention Study Family (ASPS-Fam): The authors thank the staff and the participants for their valuable contributions. We thank Birgit Reinhart for her long-term administrative commitment, Elfi Hofer for the technical assistance at creating the DNA bank, Ing. Johann Semmler and Anita Harb for DNA sequencing and DNA analyses by TaqMan assays and Irmgard Poelzl for supervising the quality management processes after ISO9001 at the biobanking and DNA analyses.

The research reported in this article was funded by the Austrian Science Fund (FWF) grant number P20545-P05, P13180, PI904, P20545-B05 the Austrian National Bank Anniversary Fund, P15435, and by the Austrain Federal Ministry of Science, Research and Economy under the aegis of the EU Joint Programme-Neurodegenerative Disease Research (JPND)-www.jpnd.eu. The Medical University of Graz and the Steiermärkische Krankenanstaltengesellschaft support the databank of the ASPS.

Cardiovascular Health Study (CHS): The CHS research was supported by NHLBI contracts HHSN268201200036C, HHSN268200800007C, N01HC55222, N01HC85079, N01HC85080, N01HC85081, N01HC85082, N01HC85083, N01HC85086, N01HC15103; and NHLBI grants HL080295, HL087652, HL105756, HL103612, HL120393 and HL130114 with additional contribution from the

National Institute of Neurological Disorders and Stroke (NINDS). Additional support was provided through AG023629 and AG15928 from the National Institute on Aging (NIA). A full list of principal CHS investigators and institutions can be found at CHS-NHLBI.org/. The provision of genotyping data was supported in part by the National Center for Advancing Translational Sciences, CTSI grant UL1TR000124, and the National Institute of Diabetes and Digestive and Kidney Disease Diabetes Research Center (DRC) grant DK063491 to the Southern California Diabetes Endocrinology Research Center. The content is solely the responsibility of the authors and does not necessarily represent the official views of the National Institutes of Health.

Epidemiology of Dementia in Singapore (EDIS): The Singapore Malay Eye Study (SiMES) and the Singapore Chinese Eye. Study (SCES) are funded by National Medical Research Council (grants 0796/2003, IRG07nov013, IRG09nov014, STaR/0003/2008 and CG/SERI/2010) and Biomedical Research Council (grants 09/1/35/19/616), Singapore. The Genome Institute of Singapore, Agency for Science, Technology and Research, Singapore provided services for genotyping. The Epidemiology of Dementia in Singapore study is supported by the National Medical Research Council, Singapore (NMRC/CG/NUHS/2010 [Grant no: R-184-006-184-511]). Dr. M.K. Ikram received additional funding from the Singapore Ministry of Health's National Medical Research Council (NMRC/CSA/038/2013).

Erasmus Rucphen family study (ERF): The ERF study as a part of EUROSPAN (European Special Populations Research Network) was supported by European Commission FP6 STRP grant number 018947 (LSHG-CT-2006-01947) and also received funding from the European Community's Seventh Framework Programme (FP7/2007-2013)/grant agreement HEALTH-F4-2007-201413 by the European Commission under the programme "Quality of Life and Management of the Living Resources" of 5th Framework Programme (no. QLG2-CT-2002-01254). High-throughput analysis of the ERF data was supported by a joint grant from the Netherlands Organization for Scientific Research and the Russian Foundation for Basic Research (NWO-RFBR 047.017.043). Najaf Amin is supported by the Netherlands Brain Foundation (project number F2013(1)-28). We are grateful to all study participants and their relatives, general practitioners and neurologists for their contributions and to P. Veraart for her help in genealogy, J. Vergeer for the supervision of the laboratory work and P. Snijders for his help in data collection.

Framingham Heart Study (FHS): The Framingham Heart Study is supported by the National Heart, Lung and Blood Institute (NHLBI) Framingham Heart Study (contracts N01-HC-25195 and HHSN268201500001I), the Boston University School of Medicine, and by grants from the National Institute of Aging (AG033040, AG033193, AG054076, AG049607, AG008122) and the National Institute of Neurological Disorders and Stroke (NS017950). We would like to thank the dedication of

the Framingham Study participants. We also thank the Framingham Study team, especially the Neurology core, both investigators and staff, for their contributions to data collection.

Genetic Study of Atherosclerosis Risk (GeneSTAR): is supported by grants from the National Institutes of Health National Institute of Neurological Disorders and Stroke (R01NS062059), the National Institutes of Health National Heart, Lung, and Blood Institute (U01 HL72518, HL097698) and the National Institutes of Health/National Center for Research Resources (M01-RR000052) to the Johns Hopkins General Clinical Research Center. We would like to thank the participants and families of GeneSTAR and our dedicated staff for all their sacrifices.

Leiden Longevity study (LLS): The research leading to these results has received funding from the European Union's Seventh Framework Programme (FP7/2007-2011) under grant agreement number 259679. This study was financially supported by the Innovation-Oriented Research Program on Genomics (SenterNovem IGE05007), the Centre for Medical Systems Biology and the Netherlands Consortium for Healthy Ageing (grant 050-060-810), all in the framework of the Netherlands Genomics Initiative, Netherlands Organization for Scientific Research (NWO)by BBMRI-NL, a Research Infrastructure financed by the Dutch government (NWO 184.021.007), and by Unilever Colworth.

Religious Orders Study and Memory and Aging Project (ROSMAP): The clinical, genomic, and neuroimaging data for the Religious Orders Study and the Rush Memory and Aging Project was funded by NIH grants P30AG10161, RF1AG15819, R01AG17917, R01AG30146, R01AG40039, and the Translational Genomics Research Institute.

Rotterdam Study (RSI, RSII, RSIII): Rotterdam Study (RS): The Rotterdam Study is funded by Erasmus Medical Center and Erasmus University, Rotterdam, Netherlands Organization for the Health Research and Development (ZonMw), the Research Institute for Diseases in the Elderly (RIDE), the Ministry of Education, Culture and Science, the Ministry for Health, Welfare and Sports, the European Commission (DG XII), and the Municipality of Rotterdam. The authors are grateful to the study participants, the staff from the Rotterdam Study and the participating general practitioners and pharmacists. The generation and management of GWAS genotype data for the Rotterdam Study (RS I, RS II, RS III) were executed by the Human Genotyping Facility of the Genetic Laboratory of the Department of Internal Medicine, Erasmus MC, Rotterdam, The Netherlands. The GWAS datasets are supported by the Netherlands Organisation of Scientific Research NWO Investments (nr. 175.010.2005.011, 911-03-012), the Genetic Laboratory of the Department of Internal Medicine, Erasmus MC, the Research Institute for Diseases in the Elderly (014-93-015; RIDE2), the Netherlands Genomics Initiative (NGI)/Netherlands Organisation for Scientific Research (NWO) Netherlands Consortium for Healthy Aging (NCHA), project nr. 050-060-810. We thank Pascal Arp, Mila Jhamai,

Marijn Verkerk, Lizbeth Herrera and Marjolein Peters, and Carolina Medina-Gomez, for their help in creating the GWAS database, and Karol Estrada, Yurii Aulchenko, and Carolina Medina-Gomez, for the creation and analysis of imputed data. This work has been performed as part of the CoSTREAM project (www.costream.eu) and has received funding from the European Union's Horizon 2020 research and innovation programme under grant agreement No 667375. HHHA is supported by ZonMW grant number 916.19.151.

The Saguenay Youth Study (SYS): The Saguenay Youth Study has been funded by the Canadian Institutes of Health Research (TP, ZP), Heart and Stroke Foundation of Canada (ZP), and the Canadian Foundation for Innovation (ZP). We thank all families who took part in the Saguenay Youth Study. SYS is supported by the Canadian Institutes of Health Research: NET54015, NRF86678, TMH109788.

Three City Study (3C-Dijon): The 3-City Study is conducted under a partnership agreement among the Institut National de la Santé et de la Recherche Médicale (INSERM), the University of Bordeaux, and Sanofi-Aventis. The Fondation pour la Recherche Médicale funded the preparation and initiation of the study. The 3C Study is also supported by the Caisse Nationale Maladie des Travailleurs Salariés, Direction Générale de la Santé, Mutuelle Générale de l'Education Nationale (MGEN), Institut de la Longévité, Conseils Régionaux of Aquitaine and Bourgogne, Fondation de France, and Ministry of Research–INSERM Programme "Cohortes et collections de données biologiques." This work was supported by the National Foundation for Alzheimer's Disease and Related Disorders, INSERM, FRC, Rotary, Lille Génopôle, the Institut Pasteur de Lille, the Centre National de Génotypage, the University of Lille, the Centre Hospitalier Universitaire de Lille, and the LABEX DISTALZ (Development of Innovative Strategies for a Transdisciplinary approach to ALZheimer's disease). Stéphanie Debette is recipient of a grant from the French National Research Agency (ANR), the Joint Programme for Neurodegenerative Disease Research (JPND), the European Research Council (ERC).

UK Biobank (UKBB)

This research has been conducted using the UK Biobank Resource under Application Number 23509.

References

1. Vrooman, H.A., *et al.* Multi-spectral brain tissue segmentation using automatically trained k-Nearest-Neighbor classification. *Neuroimage* **37**, 71-81 (2007).
2. Harris, T.B., *et al.* Age, Gene/Environment Susceptibility-Reykjavik Study: multidisciplinary applied phenomics. *American journal of epidemiology* **165**, 1076-1087 (2007).
3. Sigurdsson, S., *et al.* Brain tissue volumes in the general population of the elderly: the AGES-Reykjavik study. *Neuroimage* **59**, 3862-3870 (2012).
4. Forsberg, L., *et al.* The AGES-Reykjavik study atlases: Non-linear multi-spectral template and atlases for studies of the ageing brain. *Med Image Anal* **39**, 133-144 (2017).
5. The Atherosclerosis Risk in Communities (ARIC) Study: design and objectives. The ARIC investigators. *American journal of epidemiology* **129**, 687-702 (1989).
6. Howard, G., *et al.* Cigarette smoking and other risk factors for silent cerebral infarction in the general population. *Stroke* **29**, 913-917 (1998).
7. Bryan, R.N., *et al.* A method for using MR to evaluate the effects of cardiovascular disease on the brain: the cardiovascular health study. *AJNR Am J Neuroradiol* **15**, 1625-1633 (1994).
8. Seiler, S., *et al.* Magnetization transfer ratio relates to cognitive impairment in normal elderly. *Front Aging Neurosci* **6**, 263 (2014).
9. Ghadery, C., *et al.* R2* mapping for brain iron: associations with cognition in normal aging. *Neurobiol Aging* **36**, 925-932 (2015).
10. Schmidt, R., Fazekas, F., Kapeller, P., Schmidt, H. & Hartung, H.P. MRI white matter hyperintensities: three-year follow-up of the Austrian Stroke Prevention Study. *Neurology* **53**, 132-139 (1999).
11. Schmidt, R., *et al.* Assessment of cerebrovascular risk profiles in healthy persons: definition of research goals and the Austrian Stroke Prevention Study (ASPS). *Neuroepidemiology* **13**, 308-313 (1994).
12. Fried, L.P., *et al.* The Cardiovascular Health Study: design and rationale. *Ann Epidemiol* **1**, 263-276 (1991).
13. Hilal, S., *et al.* Prevalence of cognitive impairment in Chinese: epidemiology of dementia in Singapore study. *Journal of neurology, neurosurgery, and psychiatry* **84**, 686-692 (2013).
14. Cornes, B.K., *et al.* Identification of four novel variants that influence central corneal thickness in multi-ethnic Asian populations. *Hum Mol Genet* **21**, 437-445 (2012).
15. Vithana, E.N., *et al.* Collagen-related genes influence the glaucoma risk factor, central corneal thickness. *Hum Mol Genet* **20**, 649-658 (2011).
16. Ibrahim-Verbaas, C.A., *et al.* Linkage analysis for plasma amyloid beta levels in persons with hypertension implicates A beta-40 levels to presenilin 2. *Hum Genet* **131**, 1869-1876 (2012).
17. Ikram, M.A., *et al.* The Rotterdam Scan Study: design update 2016 and main findings. *Eur J Epidemiol* **30**, 1299-1315 (2015).
18. Dawber, T.R. & Kannel, W.B. The Framingham study. An epidemiological approach to coronary heart disease. *Circulation* **34**, 553-555 (1966).
19. Feinleib, M., Kannel, W.B., Garrison, R.J., McNamara, P.M. & Castelli, W.P. The Framingham Offspring Study. Design and preliminary data. *Prev Med* **4**, 518-525 (1975).
20. Splansky, G.L., *et al.* The Third Generation Cohort of the National Heart, Lung, and Blood Institute's Framingham Heart Study: design, recruitment, and initial examination. *American journal of epidemiology* **165**, 1328-1335 (2007).
21. Aljabar, P., Heckemann, R.A., Hammers, A., Hajnal, J.V. & Rueckert, D. Multi-atlas based segmentation of brain images: atlas selection and its effect on accuracy. *Neuroimage* **46**, 726-738 (2009).
22. Rueckert, D., Aljabar, P., Heckemann, R.A., Hajnal, J.V. & Hammers, A. Diffeomorphic registration using B-splines. *Med Image Comput Comput Assist Interv* **9**, 702-709 (2006).

23. Kochunov, P.*, et al.* Regional spatial normalization: toward an optimal target. *J Comput Assist Tomogr* **25**, 805-816 (2001).
24. Fletcher, E., Carmichael, O. & Decarli, C. MRI non-uniformity correction through interleaved bias estimation and B-spline deformation with a template. *Conference proceedings : ... Annual International Conference of the IEEE Engineering in Medicine and Biology Society. IEEE Engineering in Medicine and Biology Society. Conference* **2012**, 106-109 (2012).
25. Rajapakse, J.C.*, et al.* A technique for single-channel MR brain tissue segmentation: application to a pediatric sample. *Magn Reson Imaging* **14**, 1053-1065 (1996).
26. Fletcher, E., Singh, B., Harvey, D., Carmichael, O. & Decarli, C. Adaptive image segmentation for robust measurement of longitudinal brain tissue change. *Conference proceedings : ... Annual International Conference of the IEEE Engineering in Medicine and Biology Society. IEEE Engineering in Medicine and Biology Society. Conference* **2012**, 5319-5322 (2012).
27. Schoenmaker, M.*, et al.* Evidence of genetic enrichment for exceptional survival using a family approach: the Leiden Longevity Study. *Eur J Hum Genet* **14**, 79-84 (2006).
28. Westendorp, R.G.*, et al.* Nonagenarian siblings and their offspring display lower risk of mortality and morbidity than sporadic nonagenarians: The Leiden Longevity Study. *J Am Geriatr Soc* **57**, 1634-1637 (2009).
29. Fleischman, D.A.*, et al.* Faster cognitive decline in the years prior to MR imaging is associated with smaller hippocampal volumes in cognitively healthy older persons. *Front Aging Neurosci* **5**, 21 (2013).
30. Pausova, Z.*, et al.* Cohort Profile: The Saguenay Youth Study (SYS). *Int J Epidemiol* **46**, e19 (2017).
31. Sled, J.G., Zijdenbos, A.P. & Evans, A.C. A nonparametric method for automatic correction of intensity nonuniformity in MRI data. *IEEE Trans Med Imaging* **17**, 87-97 (1998).
32. Zijdenbos, A.P., Forghani, R. & Evans, A.C. Automatic "pipeline" analysis of 3-D MRI data for clinical trials: application to multiple sclerosis. *IEEE Trans Med Imaging* **21**, 1280-1291 (2002).
33. Avants, B.B., Epstein, C.L., Grossman, M. & Gee, J.C. Symmetric diffeomorphic image registration with cross-correlation: evaluating automated labeling of elderly and neurodegenerative brain. *Med Image Anal* **12**, 26-41 (2008).
34. Grabner, G.*, et al.* Symmetric atlasing and model based segmentation: an application to the hippocampus in older adults. *Med Image Comput Comput Assist Interv* **9**, 58-66 (2006).
35. Tohka, J., Zijdenbos, A. & Evans, A. Fast and robust parameter estimation for statistical partial volume models in brain MRI. *Neuroimage* **23**, 84-97 (2004).
36. Collins, D.L., Holmes, C.J., Peters, T.M. & Evans, A.C. Automatic 3-D model-based neuroanatomical segmentation. *Human Brain Mapping* **3**, 190-208 (1995).
37. Group, C.S. Vascular factors and risk of dementia: design of the Three-City Study and baseline characteristics of the study population. *Neuroepidemiology* **22**, 316-325 (2003).
38. Godin, O.*, et al.* White matter lesions as a predictor of depression in the elderly: the 3C-Dijon study. *Biological psychiatry* **63**, 663-669 (2008).
39. Soumare, A.*, et al.* White matter lesions volume and motor performances in the elderly. *Ann Neurol* **65**, 706-715 (2009).
40. Bis, J.C.*, et al.* Common variants at 12q14 and 12q24 are associated with hippocampal volume. *Nat Genet* **44**, 545-551 (2012).
41. Lambert, J.C.*, et al.* Genome-wide association study identifies variants at CLU and CR1 associated with Alzheimer's disease. *Nat Genet* **41**, 1094-1099 (2009).
42. Bycroft, C.*, et al.* Genome-wide genetic data on ~500,000 UK Biobank participants. *bioRxiv* (2017).
43. Alfaro-Almagro, F.*, et al.* Image Processing and Quality Control for the first 10,000 Brain Imaging Datasets from UK Biobank. *bioRxiv* (2017).
44. Miller, K.L.*, et al.* Multimodal population brain imaging in the UK Biobank prospective epidemiological study. *Nat Neurosci* **19**, 1523-1536 (2016).

45. Bulik-Sullivan, B.K., *et al.* LD Score regression distinguishes confounding from polygenicity in genome-wide association studies. *Nat Genet* **47**, 291-295 (2015).
