# Supplementary material for: A genome-wide association study identifies genetic loci associated with specific lobar brain volumes
Source: Commun Biol. 2019 Aug 2;2:285. doi: 10.1038/s42003-019-0537-9 (PMC6677735; doi:10.1038/s42003-019-0537-9)
Supplement: Supplementary file 2 — Description of Supplementary Data [file 42003_2019_537_MOESM2_ESM.docx]

**Supplementary Data 1:** Study descriptive statistics.

**Supplementary Data 2:** Description of the genotyping used and imputation QC metrics by study.

**Supplementary Data 3:** Summary statistics of the lobar brain volumes by study.

**Supplementary Data 4:** Number of variants and inflation factor by study and by lobar volume analysis. Annotation based on HaploReg.^28^

**Supplementary Data 5:** Heritability of lobar brain volumes in the Framingham Heart Study.

**Supplementary Data 6:** Variants genome-wide significantly associated with brain lobar volumes.

**Supplementary Data 7:** The association results of the lead SNPs with all brain lobar volumes.

**Supplementary Data 8:** Percentage of the heritability explained by common SNPs.

**Supplementary Data 9:** Genetic correlations of lobar volumes with lobar volumes, lobar volumes with other brain-related traits and lobar volumes with neurological and psychiatric diseases.
