# Supplementary material for: A genome-wide association study identifies genetic loci associated with specific lobar brain volumes
Source: Commun Biol. 2019 Aug 2;2:285. doi: 10.1038/s42003-019-0537-9 (PMC6677735; doi:10.1038/s42003-019-0537-9)
Supplement: Supplementary file 4 — Reporting Summary [file 42003_2019_537_MOESM4_ESM.pdf]

nature research

Corresponding author(s): Hieab H.H. Adams & Charles DeCarli

Reporting Summary

Nature Research wishes to improve the reproducibility of the work that we publish. This form provides structure for consistency and transparency in reporting. For further information on Nature Research policies, see Authors & Referees and the Editorial Policy Checklist.

Statistical parameters

When statistical analyses are reported, confirm that the following items are present in the relevant location (e.g. figure legend, table legend, main text, or Methods section).

n/a	Confirmed	
☐	☒	The exact sample size (n) for each experimental group/condition, given as a discrete number and unit of measurement
☐	☒	An indication of whether measurements were taken from distinct samples or whether the same sample was measured repeatedly
☐	☒	The statistical test(s) used AND whether they are one- or two-sided<br>*Only common tests should be described solely by name; describe more complex techniques in the Methods section.*
☐	☒	A description of all covariates tested
☐	☒	A description of any assumptions or corrections, such as tests of normality and adjustment for multiple comparisons
☐	☒	A full description of the statistics including central tendency (e.g. means) or other basic estimates (e.g. regression coefficient) AND variation (e.g. standard deviation) or associated estimates of uncertainty (e.g. confidence intervals)
☐	☒	For null hypothesis testing, the test statistic (e.g. F, t, r) with confidence intervals, effect sizes, degrees of freedom and P value noted<br>*Give P values as exact values whenever suitable.*
☒	☐	For Bayesian analysis, information on the choice of priors and Markov chain Monte Carlo settings
☒	☐	For hierarchical and complex designs, identification of the appropriate level for tests and full reporting of outcomes
☐	☒	Estimates of effect sizes (e.g. Cohen's d, Pearson's r), indicating how they were calculated
☐	☒	Clearly defined error bars<br>*State explicitly what error bars represent (e.g. SD, SE, CI)*

Our web collection on statistics for biologists may be useful.

Software and code

Policy information about availability of computer code

Data collection	No software was used for the data collection of this study.

Data analysis	Software used for the data analysis of this study:<br>EasyQC (www.genepi-regensburg.de/easyqc)<br>FreeSurfer (https://surfer.nmr.mgh.harvard.edu/)<br>GCTA (http://cnsgenomics.com/software/gcta/)<br>GenABEL (http://www.genabel.org)<br>GTeX (https://gtexportal.org/home/)<br>HaploReg (https://www.encodeproject.org/software/haploreg/)<br>HASE (https://github.com/roshchupkin/hase)<br>LD-hub (http://ldsc.broadinstitute.org/ldhub/)<br>LD score regression (https://github.com/bulik/ldsc)<br>mach2qtl (https://www.nitrc.org/projects/mach2qtl/)<br>METAL (http://csg.sph.umich.edu/abecasis/metal/)<br>Perl (https://www.perl.org/)<br>PLINK (https://www.cog-genomics.org/plink2)<br>R (https://www.r-project.org/)<br>SNPTEST (https://mathgen.stats.ox.ac.uk/genetics_software/snptest/snptest.html)<br>SOLAR (http://www.sfbr.org)

For manuscripts utilizing custom algorithms or software that are central to the research but not yet described in published literature, software must be made available to editors/reviewers upon request. We strongly encourage code deposition in a community repository (e.g. GitHub). See the Nature Research guidelines for submitting code & software for further information.

Data

Policy information about availability of data

All manuscripts must include a data availability statement. This statement should provide the following information, where applicable:

- Accession codes, unique identifiers, or web links for publicly available datasets
- A list of figures that have associated raw data
- A description of any restrictions on data availability

The genome-wide summary statistics that support the findings of this study are available upon request from the corresponding authors HHHA and CD.

Field-specific reporting

Please select the best fit for your research. If you are not sure, read the appropriate sections before making your selection.

☒ Life sciences ☐ Behavioural & social sciences ☐ Ecological, evolutionary & environmental sciences

For a reference copy of the document with all sections, see nature.com/authors/policies/ReportingSummary-flat.pdf

Life sciences study design

All studies must disclose on these points even when the disclosure is negative.

Sample size	No sample size calculation was performed. We aimed to include all cohorts with available data for brain lobar volumes.
Data exclusions	Individuals with dementia or stroke were excluded since these disorders can have a substantial influence on lobar volume measurements.
Replication	We were able to replicate our findings in an independent population-based cohort study (N=8,789).
Randomization	This is not relevant to our study since no randomization was performed.
Blinding	This is not relevant to our study since no blinding was performed.

Reporting for specific materials, systems and methods

Materials & experimental systems

n/a	Involved in the study
☒	☐ Unique biological materials
☒	☐ Antibodies
☒	☐ Eukaryotic cell lines
☒	☐ Palaeontology
☒	☐ Animals and other organisms
☐	☒ Human research participants

Methods

n/a	Involved in the study
☒	☐ ChIP-seq
☒	☐ Flow cytometry
☐	☒ MRI-based neuroimaging

Human research participants

Policy information about studies involving human research participants

Population characteristics: The study sample consisted of dementia-free and stroke-free individuals with quantitative brain MRI and genome-wide genotypes. Studies with varying study designs and population characteristics were included, as shown in Supplementary Data 1.

Recruitment: The recruitment of the studies differed; for more information please refer to the Supplementary information.

Magnetic resonance imaging

Experimental design

Design type: This is not relevant to our study since no fMRI data was used.

Design specifications: This is not relevant to our study since no fMRI data was used.

Behavioral performance measures: This is not relevant to our study since no fMRI data was used.

Acquisition

Imaging type(s): For this study, structural MRI was used.

Field strength: MRI scanners with different field strengths were used for this study, both 1.5 and 3T (Supplementary Table 1).

Sequence & imaging parameters: The sequence and imaging parameters varied for the different study samples. For a description, see the Supplementary information.

Area of acquisition: The whole brain scan was used to measure brain lobar volumes.

Diffusion MRI ☐ Used ☒ Not used

Preprocessing

Preprocessing software: The preprocessing varied for the different study samples. For a description, see the Supplementary information.

Normalization: The normalization varied for the different study samples. For a description, see the Supplementary information.

Normalization template: The normalization template varied for the different study samples. For a description, see the Supplementary information.

Noise and artifact removal: The noise and artifact removal varied for the different study samples. For a description, see the Supplementary information.

Volume censoring: The volume censoring varied for the different study samples. For a description, see the Supplementary information.

Statistical modeling & inference

Model type and settings: Associations of imputed genotype dosages with lobar volumes were examined using linear regression analyses (or linear mixed models for family studies) under an additive model, adjusted for age, age^2, sex, the first four principal components to account for possible confounding due to population stratification, and study-specific covariates.

Effect(s) tested: We tested the effect of an increase in an effect allele on the volumes of the four human brain lobes.

Specify type of analysis: ☐ Whole brain ☒ ROI-based ☐ Both

Anatomical location(s): Frontal lobe, temporal lobe, parietal lobe, occipital lobe.

Statistic type for inference (See Eklund et al. 2016): T-values/p-values obtained from linear regression analyses (or linear mixed models for studies with related individuals)

Correction

We used the accepted standard p-value threshold for significance for genome-wide association studies (5e-08) and we have replicated the findings afterwards.

Models & analysis

n/a	Involved in the study
☒	☐ Functional and/or effective connectivity
☒	☐ Graph analysis
☒	☐ Multivariate modeling or predictive analysis
